# Mechanistic Insights into the Suppression of Proton Intercalation and the Hydrogen Evolution Reaction through Phosphorus Doping in Tungsten Oxide

**DOI:** 10.1021/acselectrochem.6c00059

**Published:** 2026-05-06

**Authors:** Oguz Kaan Kucukosman, Hengfei Gu, Xueyuan Zhang, Jacob Smith, Zhiyuan Zhang, Elizabeth Desmet, Yiguang Ju, Eric Garfunkel, Miaofang Chi, Aditya Dilip Lele, Huixin He

**Affiliations:** † Department of Chemistry, 242612 Rutgers, the State University of New Jersey, Newark 07102, New Jersey, United States; ‡ Department of Chemistry and Chemical Biology, 67206 Rutgers University, New Brunswick 08901, New Jersey, United States; § 440052 Gamry Instruments, Inc., Warminster 18974, Pennsylvania, United States; ∥ Center for Nanophase Materials Sciences, Oak Ridge National Laboratory, Oak Ridge 37831, Tennessee, United States; ⊥ Department of Mechanical and Aerospace Engineering, 6740 Princeton University, Princeton 08544, New Jersey, United States; # Thomas Lord Department of Mechanical Engineering & Materials Science, 3065 Duke University, Durham 27708, North Carolina, United States; ¶ Department of Mechanical Engineering, 3536 Rowan University, Glassboro 08028, New Jersey, United States

**Keywords:** proton intercalation, HER suppression, phosphorus doping, WO_3_, transition metal oxides

## Abstract

The hydrogen evolution reaction (HER) is an inevitable parasitic process that limits the efficiency and selectivity of electrochemical hydrogenation reactions using water as the hydrogen source. Although introducing oxygen vacancies and heteroatom dopants into transition-metal oxides is widely employed to enhance hydrogenation activity, such modifications often inadvertently promote HER. Here, we demonstrate a counterintuitive suppression of proton intercalation and HER in metalloid phosphorus (P)-doped WO_3_ catalysts, with progressively stronger suppression at higher P-doping levels. Electrochemical impedance spectroscopy, Mott–Schottky analysis, and hydrogen bond dissociation free energy (H-BDFE) measurement reveal that, despite enhanced electronic conductivity, improved interfacial charge transfer, and decreased H-BDFE, phosphorus doping significantly increases the adsorption resistance associated with W–H* intermediate formation and reduces H* surface coverage, thereby suppressing HER kinetics. Density functional theory calculations further show that even though the W d-band center was downshifted toward its Fermi level, P-doping broadens the distribution of hydrogen binding strengths across oxygen sites of the WO_3_ catalysts, such that many sites bind hydrogen too weakly to support efficient proton intercalation. These insights reveal an alternative HER suppression mechanism whereby heteroatom doping enables local control of proton intercalation and hydrogen adsorption kinetics beyond conventional d-band tuning, proton/electron supply, or charge-transport limitations.

## Graphical abstract

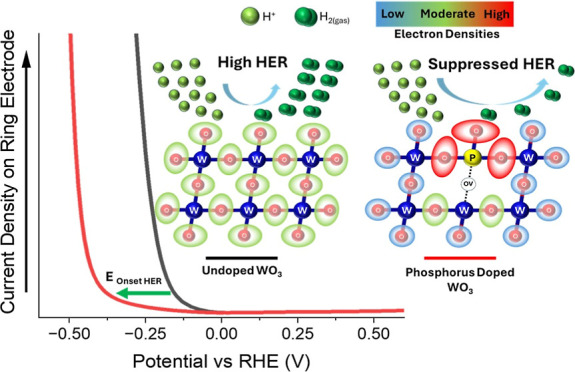

## Introduction

The hydrogen evolution reaction (HER) is a key cathodic process in electrochemical water splitting for H_2_ production, providing a high-gravimetric-energy-density fuel and a valuable hydrogen source for various chemical syntheses, particularly hydrogenation reactions. Electrochemical hydrogenation, which directly uses water as the hydrogen source and thereby avoids challenges associated with H_2_ storage, transportation, and safety, offers a promising route for converting abundant and thermodynamically stable molecules, such as nitrogen (N_2_)^1–3^ and carbon dioxide (CO_2_)^1,2^ into value-added chemicals and fuels using renewable electricity. However, HER acts as a parasitic competing reaction in these systems, narrowing the useable electrochemical window and hindering the desired reduction pathways.^1–8^ In fact, the inevitably competing HER has been the major obstacle impeding the practical implementation of these electrochemical hydrogenation technologies, as it significantly lowers attainable product (FE).^1–8^

Significant efforts have been devoted to suppressing HER to enhance the efficiency and selectivity of these electrocatalytic hydrogenation reactions.^8–200^ However, it is fundamentally challenging to develop catalysts that simultaneously promote the desired hydrogenation reduction while suppressing HER, due to the scaling relationships governing the adsorption energies of key reaction intermediates.^8–10^ Using electrocatalytic nitrogen reduction (eNRR) to NH_3_ as an example, most catalysts (except for some single-atom-based systems) that bind N_2_ and nitrogen-containing intermediates strongly enough to activate N_2_ also tend to bind H strongly, thereby favoring the competing hydrogen evolution reaction (HER).^1,10–13^

Prior research efforts have also focused on suppressing HER from a kinetic perspective.^7–9^ Nørskov and co-workers developed a qualitative model showing that, beyond a certain regime, the NH_3_ production rate is not directly correlated with proton or electron concentrations, whereas H_2_ formation exhibits first-order dependence on both.^14^ In other words, limiting the availability of either protons or electrons can efficiently suppress the HER without significantly affecting NH_3_ production within a certain range. On this basis, kinetic strategies for HER suppression have been developed by restricting proton and electron accessibility during the eNRR. For example, increasing the electrolyte pH^15–20^ or employing organic–aqueous hybrid electrolytes reduces proton concentration,^21–24^ while the addition of alkali metal cations decreases proton transfer to the electric double layer (EDL) at the catalyst–electrolyte interface.^25–27^ Similarly, hydrophobic surface layers can slow the transport of H_2_O (the proton donor) via hydrophobic effects.^3,28^ Strategies to limit electron accessibility include the use of catalyst materials with intrinsically low electronic conductivity or low-conductivity catalyst supports.^29–31^

Transition metal oxides (TMOs) are widely regarded as catalytically inert toward HER,^32^ primarily because of their low electronic conductivity and unfavorable hydrogen adsorption energetics,^32,33^ rendering them promising catalysts or supports for selective eNRR. Unfortunately, the eNRR performances of most reported TMOs are still far from the level required for practical implementation primarily due to their insufficient N_2_ adsorption and activation at catalytic centers. To improve the catalytic activity of TMOs for NRR, intentional introduction of structural defects, such as oxygen vacancies (OVs), and substitutional doping of heteroatoms in TMO have been demonstrated as effective strategies to promote the intrinsic activity of the catalytic centers, the number of catalytic centers,^34–36^ and therefore the overall catalytic performance.^37^

However, it is worth mentioning that the electronic and geometric changes caused by the introduction of OVs and heteroatoms into the TMO matrix may also lead to a simultaneous increase in HER activity. According to the d-band center theory,^38^ shifting the d-band center toward the Fermi energy level (*E*_F_) generally strengthens hydrogen atom (H*) adsorption on catalytic sites. It has been demonstrated that introducing oxygen vacancies downshifts the W d-band center toward the Fermi level, endowing WO_3_ with high conductivity, more active sites, and enhanced H* adsorption to desirable Δ*G*_H*_ (close to zero), thereby significantly improving its electrocatalytic activity for HER.^39,40^ The HER activity for some of the modified WO_3_ has been shown to be as high as that of commercial Pt-based catalysts, indicated by the positive onset potential for HER (0.05 V vs RHE and a Tafel slope of 38 mV dec^–1^), very close to the onset potential achieved by the commercial Pt/C catalyst for HER (30 mV dec^–1^).^39,40^

In our recent studies, we developed a simple gas–solid reaction approach to fabricate phosphorus (P)-doped WO_3_ nanosheets without surface condensation of element P.^41^ This doping process introduced P atoms into the WO_3_ lattice and simultaneously generated a substantial number of OVs. The resulting material is therefore denoted as P-OV-WO_3_. We found that the d-band center of the W atoms in P-OV-WO_3_ shifts downward, moving closer to *E*_F_ upon P-doping. As expected, this shift markedly improved electrical conductivity and N_2_ reduction activity to NH_3_, delivering a nearly 20-fold increase in NH_3_ yield compared with the undoped control WO_3_ (C-WO_3_). On the other hand, this shift would typically be expected to enhance H* adsorption at W sites, thus promoting HER activity.^39,40^ However, contrary to this expectation, our results revealed that both proton intercalation and HER were significantly inhibited in P-OV-WO_3_. While this is a desired property for selective electrocatalytic hydrogenation reactions,^1–8^ these results stand in sharp contrast to the HER behavior of previously reported oxygen-vacancy-rich WO_3_ catalysts, in which the introduction of oxygen vacancies increases electrical conductivity and significantly promotes HER activity.^39,40^ This is also a striking departure from prior theoretically proposed and experimentally demonstrated HER suppression strategies that rely on tuning the d-band center of catalytic sites^9^ and/or reducing the electrical conductivity of the catalyst or its support.^29–31^ All of these observations indicate that phosphorus doping in WO_3_ induces a fundamentally different HER suppression pathway.

To elucidate this unusual and intriguing suppression, in this work, we conducted a systematic investigation of phosphorus-doped WO_3_ electrocatalysts (P-OV-WO_3_) across a range of P-doping levels. Cyclic voltammetry (CV) was employed to examine the influence of the degree of P-doping level on proton intercalation–deintercalation and the associated HER activity. Rotating ring-disk electrode (RRDE) measurements further elucidated the relationship between proton intercalation and the HER by selectively decoupling the HER current from the total disk current, which contains contributions from both HER and proton intercalation. Additionally, the Mott Schottky study and detailed electrochemical impedance spectroscopy (EIS) studies revealed that P-OV-WO_3_ indeed exhibits higher conductivity and faster interfacial charge transfer, similar to previously reported oxygen-vacancy-rich WO_3_ catalysts.^39,40^ Furthermore, hydrogen bond dissociation free energy (H-BDFE) measurement demonstrated that the H-BDFE decreased in P-OV-WO_3_. However, the interfacial resistance for H* chemical adsorption onto the catalyst surface significantly increases, leading to a reduced surface H* coverage. The reduced availability of reactive H* intermediates on the P-OV-WO_3_ surface results in kinetic suppression of HER. Finally, first-principles density functional theory (DFT) calculations revealed that phosphorus doping changes both the electronic structure and the distribution of hydrogen binding energies on WO_3_. Specifically, P-OV-WO_3_ exhibits a lower average hydrogen binding energy and an increased spread in binding strength across oxygen sites. This leads to a scenario where many sites bind H* too weakly to populate under mild overpotentials, effectively reducing surface H* availability, even though the W d band center was downshifted toward its Fermi level and the electronic conductivity was improved. These P-doping-induced modifications to the electronic structure of WO_3_ and the associated changes in proton intercalation behavior provide a comprehensive picture of why P-OV-WO_3_ deviates from conventional HER suppression. When combined with our prior understanding of heteroatom–vacancy co-engineering for enhancing eNRR activity,^41^ the insights gained here establish a broadly applicable design principle for tuning surface reactivity to favor electrochemical hydrogenation over H_2_ evolution. These concepts extend beyond eNRR and are relevant to other proton-coupled electroreduction reactions, where suppressing HER is a key challenge for achieving high selectivity and energy efficiency.

## Experimental Section

### Fabrication of P-OV-WO_3_ and C-WO_3_ Catalysts

Phosphorus-doped WO_3_ (P-OV-WO_3_) and undoped control-WO_3_ (C-WO_3_) were synthesized using our recently developed solid–gas reaction approach rather than the commonly used solid/solid reaction route, as detailed in our prior work.^41^ Briefly, red phosphorus (RP) and tungstic acid (H_2_WO_4_, or WO_3_·H_2_O) nanosheets were used as the P-doping source and WO_3_ precursor, respectively. The H_2_WO_4_ nanosheets were first synthesized via a hydrothermal method by Nayak et al., with slight modifications.^42^ A custom-designed vertical furnace reactor was employed for the P-doping and preparation of the C-WO_3_ catalysts to prevent surface contamination by unintended elemental phosphorus condensation and deposition.^43–46^ The reactor was placed in a preheated vertical furnace at 550 °C for 20 min under an Ar-protected, closed environment. During heating, RP was evaporated at approximately 416 °C and converted to more reactive white phosphorus at even higher temperatures. Simultaneously, dehydration of H_2_WO_4_ occurs, leading to the generation of defective WO_3_, which readily reacts with white P vapor to yield the targeted P-doped WO_3_. To achieve different P-doping levels, 2 mg, 5 mg, or 7.5 mg of RP was mixed with 100 mg of H_2_WO_4_, corresponding to nominal phosphorus loadings of 2, 5, and 7.5 wt %, respectively. For the control C-WO_3_ catalysts, the synthesis was carried out under identical conditions but without the addition of red phosphorus. Thus, obtained catalysts were extensively characterized; see detailed in the following sections and Supporting Information Section S1.

### Electrochemical Measurements

All of the electrochemical measurements were performed using a CHI 760 C Potentiostat (CH Instruments, USA). Ag/AgCl (saturated KCl) and a platinum (Pt) wire were used as reference (RE) and counter electrodes (CE), respectively. To test if the Pt wire counter electrode has any potential influence on the observed proton intercalation and HER suppression, control experiments were also performed with a Ti counter electrode (Supporting Information Section S2). The applied potential measured against the Ag/AgCl reference electrode in saturated KCl was converted to the reversible hydrogen electrode (RHE) using the equation: RHE = *E*_Ag/AgCl_ (V) + 0.197V + 0.059 ×pH. Mott Schottky studies were performed to study the electron density of the P-OV-WO_3_ and C-WO_3_ catalysts, as detailed in previous work.^41^ The proton intercalation/deintercalation and HER behavior were studied via cyclic voltammetry (CV) at scan rates of 1 and 100 mV s^–1^ in an Ar-saturated electrolyte (5 mM H_2_SO_4_, pH = 2). The rotating ring-disk electrode (RRDE) experiment was conducted to identify the HER onset potential by decoupling current contributions from proton intercalation and the HER. The RRDE consisted of a 4 mm diameter glassy-carbon disk surrounded by a Pt ring, provided by ALS Co. Ltd, in Tokyo, Japan, and equipped with a RRDE-3A system from the same company. A total of 10 μL of the catalyst ink was deposited onto the disk electrode only using the same deposition process as in the CV measurements (Supporting Information Section S2.1).

The ring electrode was held at 0.89 V versus RHE to selectively oxidize the evolved hydrogen on the ring electrode, thereby isolating HER-related currents from intercalation processes occurring at the disk electrode. Linear sweeping voltammetry (LSV) has been used to control the disk potential from +1.00 to −0.50 V vs RHE at a scan rate of 10 mV·s^–1^, while disk and ring currents were concurrently recorded under a rotation rate of 1600 rpm. The measured ring currents were used to construct the corresponding Tafel plots, enabling the evaluation of HER kinetics for the different catalysts. The collection efficiency (*N*_c_) of the RRDE setup, defined as $N_{c}=\frac{I_{ring}}{I_{disk}}$ , was experimentally determined to be 0.12 under argon-purged acidic conditions (pH = 2). This efficiency factor was used to quantitatively interpret the ring current and confirm the reliability of hydrogen detection in the system.

Electrochemical impedance spectroscopy (EIS) was performed by using a Gamry Interface 1000 potentiostat to probe the kinetics of the hydrogen evolution reaction (HER) over a selected potential window. Prior to each EIS measurement, the electrode was held at the corresponding applied potential for 200 s under chronoamperometric (CA) conditions to ensure steady-state behavior. The impedance spectrum was subsequently collected over a frequency range from 10^–1^ to 10^5^ Hz. The resulting spectra were fitted using equivalent circuit models implemented in Gamry Echem Analyst (v6.33). The validity of the fitting and the physical consistency of the impedance data were verified by using the Kramers–Kronig test.

## Results and Discussion

### Structural Characterization of C-WO_3_ and P-OV-WO_3_ Catalysts

The morphology and crystallographic structures of the C-WO_3_ and P-OV-WO_3_ catalysts were characterized by scanning electron microscopy (SEM) and powder X-ray diffraction (PXRD). Representative SEM images (Figures 1a and S2) show that all P-OV-WO_3_ catalysts retain the nanoplatelet morphology of the H_2_WO_4_ precursor, whereas C-WO_3_ exhibit slightly rounded edges, consistent with our previous report.^41^ Energy-dispersive X-ray spectroscopy (EDS) as shown in Figure S3a in Supporting Information confirms successful phosphorus incorporation, while energy-dispersive X-ray mapping demonstrates a homogeneous distribution of P throughout the catalysts (Figure S3b–d).

**Figure 1:**
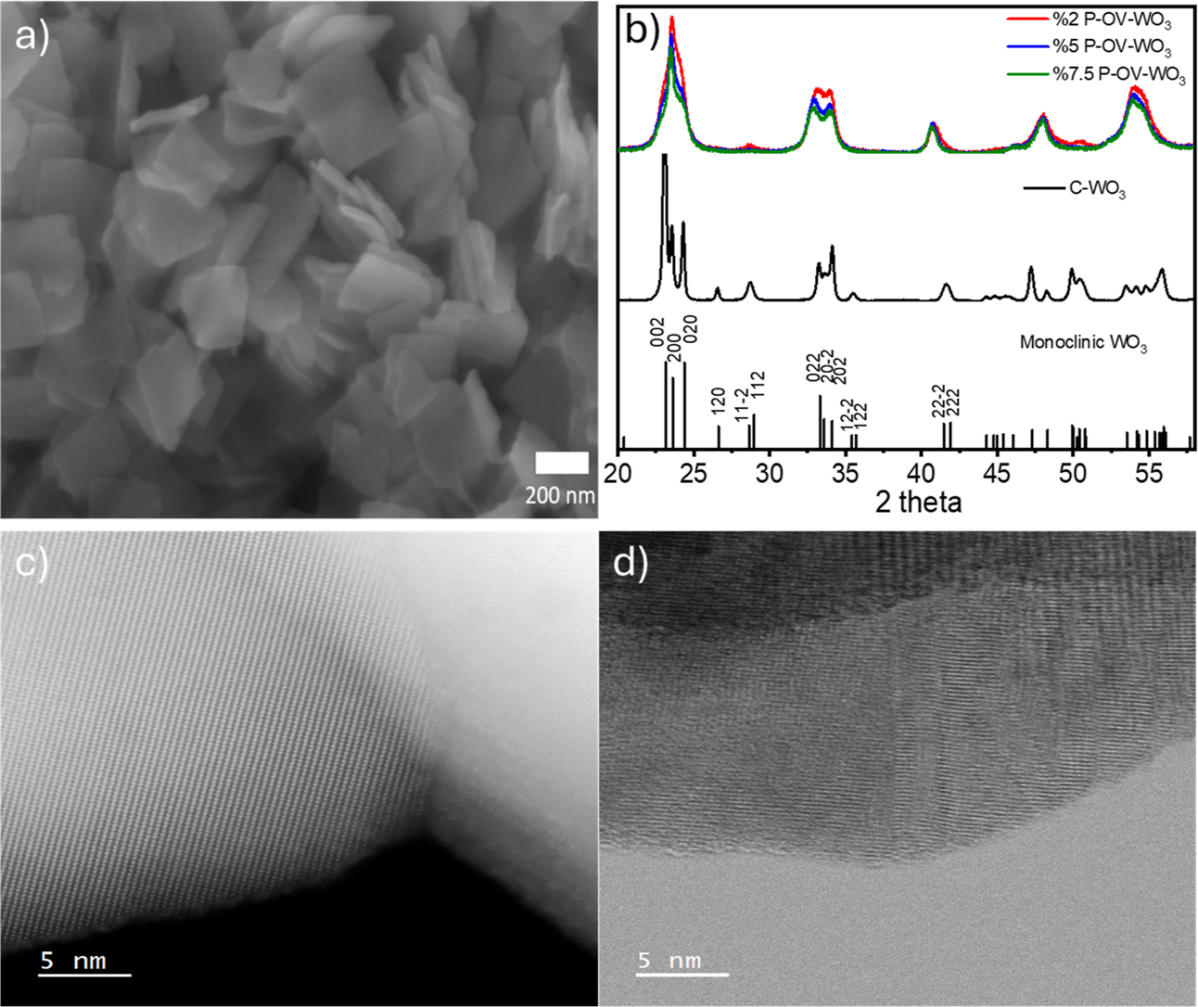
(a) SEM images of 5 at % P-OV- WO_3_, (b) PXRD spectrum of P-OV- WO_3_ catalysts, C- WO_3_, and pdf card of monoclinic WO_3_, (c) HAADF-STEM image of C-WO_3_, and (d) BF-STEM image of 5% P-OV- WO_3_.

The PXRD patterns of all samples confirm monoclinic WO_3_ as the dominant crystalline phase (Figure 1b). Upon phosphorus doping, systematic peak broadening and shifts toward lower 2θ values are observed, indicative of possible lattice distortion and defect formation. In particular, the (002) (022), and (222) reflections of P-OV-WO_3_ progressively shift to lower 2θ angles with increasing P-doping levels, corresponding to an expansion of interplanar spacing and partial amorphization. This structural evolution is consistent with the elongation of W–O bonds and reduced crystallinity, as further evidenced by decreased peak intensities and increased peak widths. Detailed analysis of lattice parameter changes and peak assignments is provided in Supporting Information Section S3.3.

To directly visualize these structural changes indicated by PXRD and EDX mapping at the atomic level, the surface structures of C-WO_3_ and P-OV-WO_3_ catalysts were further investigated using scanning transmission electron microscopy (STEM) (Figure 1c,d). In high-angle annular dark field (HAADF) STEM imaging, the contrast is dominated by atomic number (*Z*-contrast); therefore, the bright spots primarily correspond to W atomic columns, while the contributions from P and O atoms are negligible due to their much lower atomic numbers. The C-WO_3_ catalyst exhibits a highly ordered crystalline lattice with well-defined lattice fringes of 3.81 and 3.77 Å, which can be indexed to the (002) and (020) planes, respectively. The nearly perfect lattice periodicity observed in C-WO_3_ further suggests a low concentration of structural defects, consistent with its undoped, nearly stoichiometric nature. In contrast, the P-OV-WO_3_ catalyst displays a markedly different lattice structure. Although regions of crystalline order can still be indexed to the (002) and (110) planes, with lattice spacings of 3.81 and 5.45 Å, respectively, the overall lattice appears significantly distorted. As shown using bright field (BF) STEM imaging in Figure 1d, P-doping induces pronounced local strain, dislocation formation, and partially amorphous regions within the WO_3_ lattice. Consistent with the PXRD results, these structural features confirm that P-doping effectively disrupts the WO_3_ lattice, generating abundant defects and lattice strain that distinguish P-OV-WO_3_ from the highly ordered C-WO_3_ structure.

X-ray photoelectron spectroscopy (XPS) was employed to investigate the surface chemical composition and oxidation states of W and P in C-WO_3_ and P-OV-WO_3_ catalysts, providing insight into dopant-induced chemical and electronic restructuring within the near-surface region (∼10 nm in depth). The P 2p XPS spectra of all P-OV-WO_3_ catalysts display a single doublet consisting of P 2p_3/2_ and P 2p_1/2_ peaks, characteristic of P­(V) species (phosphorus in the +5-oxidation state) (Figure 2a). Notably, no signal is observed near 130 eV, indicating the absence of elemental phosphorus (*P* ^0^) in all P-doped samples. This result confirms that no condensation of *P* ^0^ occurred on the WO_3_ surface during doping.^43,47,48^

**Figure 2:**
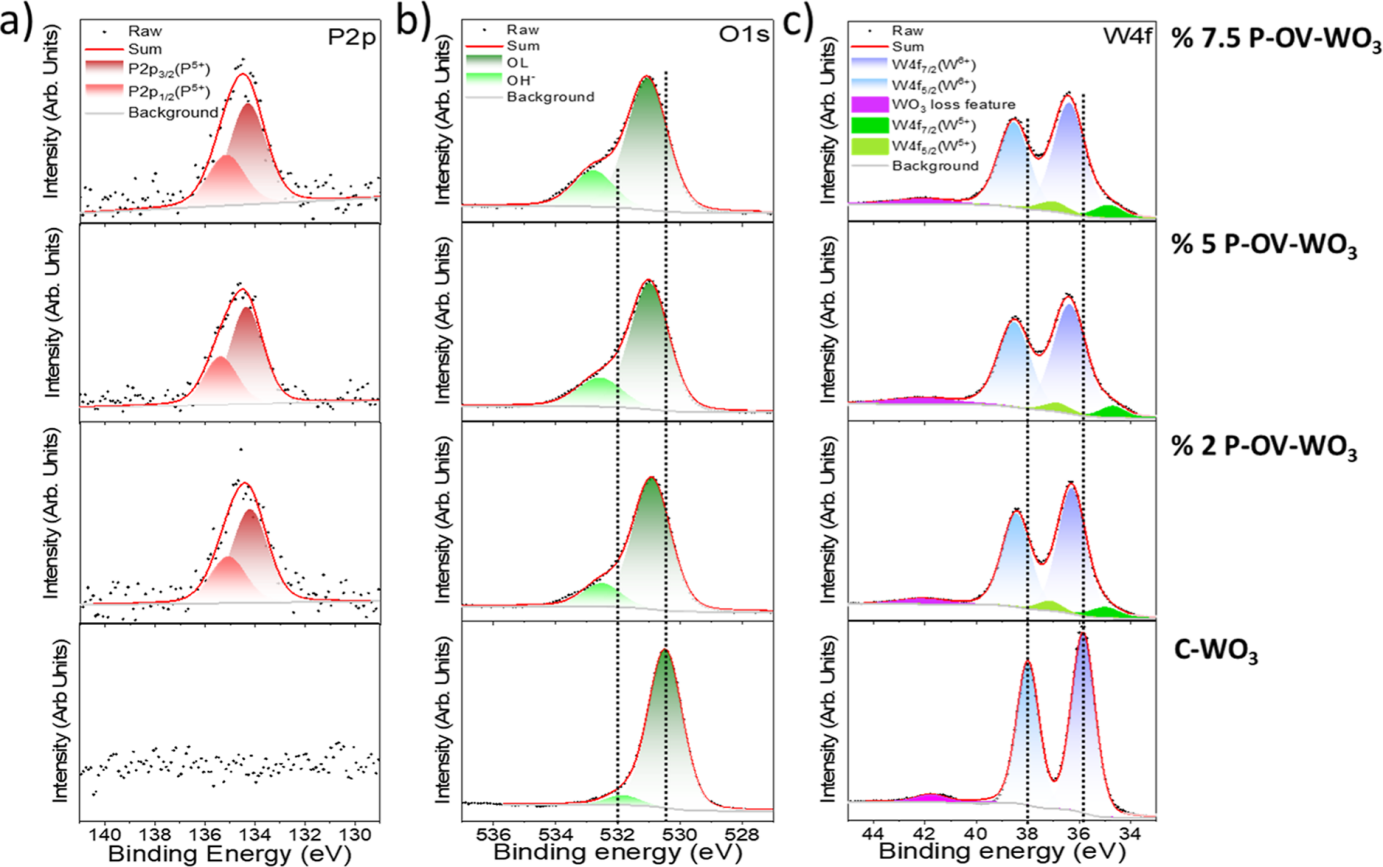
XPS spectra of (a) P 2p, (b) O 1s, (c) W 4f, C-WO_3_, and P-OV-WO_3_ catalysts.

Since P­(V) is one valence lower than W­(VI), oxygen vacancies (OVs) are introduced concurrently to maintain the charge balance during P-doping, which was also observed for cationic metallic doping.^49^ The O 1s XPS spectrum of C-WO_3_ was deconvoluted into two peaks (Figure 2b). The main peak at 531.1 eV is assigned to lattice oxygen (OL), while the higher-binding-energy peak at 532.8 eV is ascribed to surface hydroxyl species (OH^–^), arising from adsorbed water at surface and defect-rich regions, especially at OVs.^50^ With increasing P-doping levels in the P-OV-WO_3_ catalysts, the OH^–^ peak intensity increases and shifts to higher binding energies.^51^ As summarized in Tables S1 and S2, the atomic ratio of OH^–^/O (the total oxygen) calculated from their integrated XPS peak areas increases significantly from 0.065 for the control sample (C-WO_3_) to 0.275 for the 7.5% P-OV-WO_3_ catalyst, indicating a substantially high concentration of oxygen vacancies and defects induced by P-doping. Consistently, the oxygen stoichiometry derived excluding absorbed oxygen reveals increasing oxygen deficiency with P-doping levels, reaching (W/P) O_1.98_ for 7.5% P-OV-WO_3_ catalyst, compared to WO_2.25_ for C-WO_3_ (Tables S1 and S2).^50^ The existence of higher OVs and defects in P-OV-WO_3_ is consistent with the characteristic dark blue coloration of the P-OV-WO_3_ catalysts.^52^ The W 4f spectrum (Figure 2c) was deconvoluted into two doublet pairs corresponding to the W^5+^ and W^6+^ species. The P-OV-WO_3_ catalysts exhibit higher W^5+^/W^6+^ ratios than C-WO_3_, indicating that P-doping promotes the formation of reduced W­(V) centers in addition to OVs, collectively leading to increased electron density in P-OV-WO_3_ catalysts.^53^

To quantify the charge-carrier density of the catalysts, Mott–Schottky analysis was performed under dark conditions to ensure that the measured charge carriers originated from dopants and intrinsic defects rather than photoexcitation.^54^ As shown in Figure S5a in Supporting Information, all C- WO_3_ and P-OV- WO_3_ catalysts exhibit positive slopes, confirming their n-type semiconductor nature. Notably, the slopes for the P-OV-WO_3_ catalysts are approximately one order of magnitude lower than that of C-WO_3_, indicating a substantially higher electron density upon phosphorus doping. A detailed comparison of the slopes of P-OV-WO_3_ catalysts (Figure S5b) reveals a monotonic decrease with an increased P-doping level, and P-OV-WO_3_ shows increasingly metallic character in contrast with the semiconducting nature of C-WO_3_. The calculated donor densities (ND) increase from 4.91 × 10^25^ m^–3^ for C-WO_3_ to 3.86 × 10^26^, 5.38 × 10^26^, and 5.83 × 10^26^ m^–3^ for the 2%, 5%, and 7.5% P-OV-WO_3_ catalysts, respectively. These results clearly demonstrate that phosphorus doping significantly enhances the electron density within the WO_3_ lattice and with increased P-doping, and P-OV-WO_3_ shows increasingly metallic character in contrast with the semiconducting nature of C-WO_3_. In our previous work, the increased conduction-band electron density has been shown to facilitate electron donation into antibonding π* orbitals of N_2_ through π back donation, thereby enhancing N_2_ molecular activation.^41^ As noted earlier, introducing oxygen vacancies increases electron density and electrical conductivity in WO_3_, largely facilitating charge transport and proton–electron coupling and thereby enhancing HER activity.^39,40^ In the work described below, we investigate how the increased carrier density arising from combined phosphorus doping and OV introduction influences proton intercalation behavior and, in turn, HER activity in WO_3_-based catalysts.

### Effects of P-Doping on Proton Intercalation/Deintercalation and HER

As noted earlier, pristine WO_3_ is traditionally regarded as catalytically inert toward the HER due to its weak hydrogen adsorption and poor electronic conductivity. However, substantial HER currents are frequently observed when WO_3_ is employed in supercapacitors and electrochromic smart-window applications.^55,56^ This apparent contradiction has been examined in detail by the McKone and Mpourmpakis groups.^57,58^ With a combined theoretical and experimental approach, they clearly demonstrated that pristine WO_3_ is indeed not HER-active; however, WO_3_ readily undergoes bulk hydrogen intercalation in a cathodic electrochemical environment (WO_3_ + xH^+^ + xe^–^ ⇌ H_*x*_WO_3_).^56,58,59^ The in situ-achieved bulk proton intercalation enabled generation of H_*x*_WO_3_, which is likely HER-active depending on the level of proton intercalation.^57,58^ During the initial stage of intercalation, protons preferentially bind to lattice oxygen to form O–H species, while the concurrently injected electrons populate the conduction band minimum of WO_3_.^60^ Since the d orbital of the W centers is the major contributor to the bottom of the conduction band, intercalation results in electron population of the d orbital of the W centers and upshift of the Fermi level (*E*_F_). As a result, the d band center is relatively downshifted near or above its *E*_F_.^60^ Consequently, at higher cathodic potentials, continued proton–electron injection is proposed to switch hydrogen binding from lattice oxygen to W sites, leading to the formation of mobile, weakly bound hydrogen species (denoted as W–H*) in acidic electrolytes. These W–H* species have been suggested to be relatively long-lived and catalytically active toward hydrogen evolution. It was reported that significant HER is seen only at potentials more negative than the equilibrium potential required to form highly intercalated monoclinic H_*x*_WO_3_ with *x* > 0.5. In line with these studies, the Augustyn group developed approaches to modulate the HER activity of WO_3_ by introducing molecular pillars or water to suppress or facilitate proton intercalation.^61^ In contrast, the role of heteroatom doping and oxygen vacancies in regulating proton intercalation/deintercalation in WO_3_ remains largely unexplored.^41^

To probe the effect of phosphorus doping on hydrogen intercalation/deintercalation, the formation of surface-bound hydrogen intermediates (W–H*), and HER, cyclic voltammetry (CV) with a slow scan rate of 1 mV·s^–1^ was performed on all P-doped catalysts, with C-WO_3_ as a reference. As shown in Figure 3a, C-WO_3_ (black curve) exhibited three reduction peaks marked as peaks (1–3). Miu et al.^57,58^ assigned these cathodic peaks to successive proton intercalation stages corresponding to the stepwise formation of tungsten bronze phases (WO_3_ → H_0.25_WO_3_ → H_0.5_WO_3_ → H_0.625_WO_3_). They further reported that the formation of surface-bound hydrogen intermediates (W–H*) emerges upon reaching the H_0.5_WO_3_ composition within the potential window between peaks 2 and 3. After the cathodic peak 3 (∼−0.35 V vs RHE), significant HER is initiated, accompanied by observable H_2_ gas evolution at the catalyst surface. Phosphorus doping markedly alters proton interaction behavior, as evidenced by a progressive negative shift of the first proton intercalation peak accompanied by a decrease in peak current with increasing P content, indicating suppressed proton intercalation. Gaussian deconvolution of peak 1 (Figure 3b) quantifies this suppression, revealing reductions in the integrated peak area of 6%, 26%, and 38% for the 2%, 5%, and 7.5% P-OV-WO_3_ catalysts, respectively, compared to those of C-WO_3_. Notably, cathodic peaks 2 and 3, which are associated with deeper proton intercalation and the formation of W–H* intermediates, respectively, are significantly attenuated or entirely absent in the P-doped samples.

**Figure 3:**
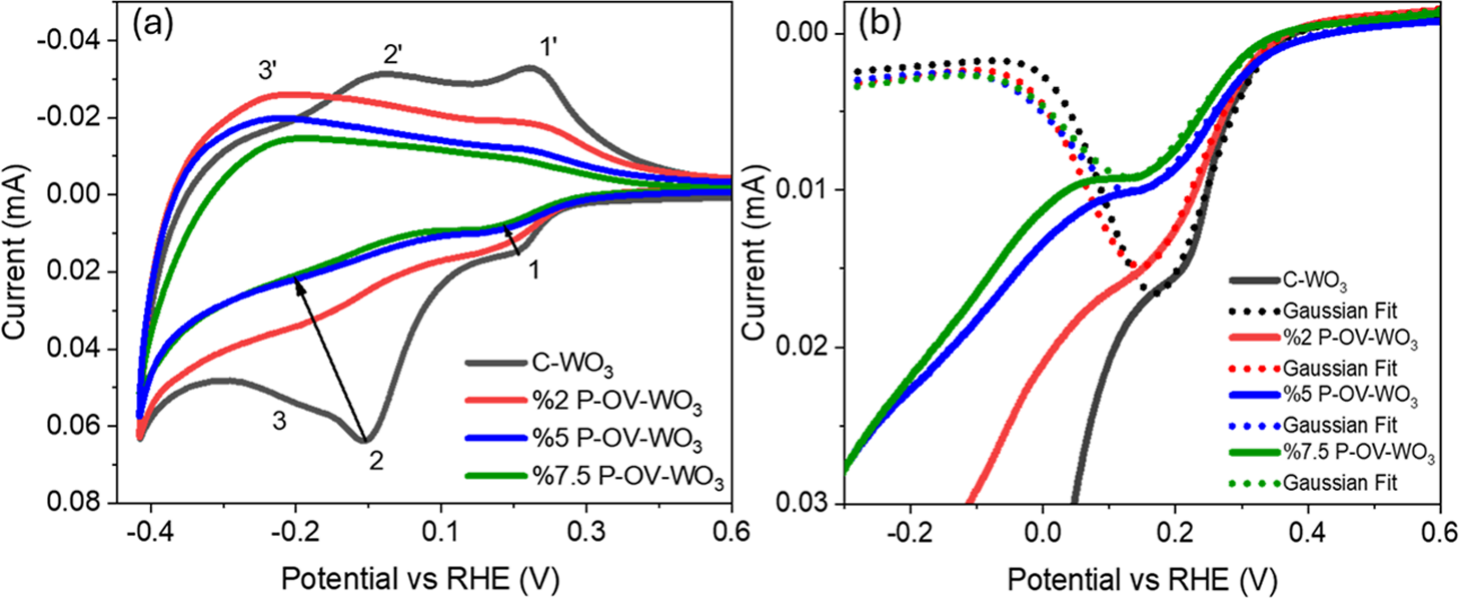
Cyclic voltammograms of all catalysts recorded over the potential range from 0.6 to −0.42 V vs RHE in an Ar-saturated pH 2 H_2_SO_4_ electrolyte, at a scan rate of 1 mV·s^–1^. (b) Enlarged view of the cathodic scan in panel (a), highlighting the first reduction peak and the corresponding Gaussian fits used to quantify and compare the integrated peak areas.

During the anodic scan, three peaks labeled 1′, 2′, and 3′ are observed (Figure 3a). Peaks 1′ and 2′ are assigned to proton deintercalation from different intercalation stages,^62^ whereas peak 3′ has not yet been unambiguously assigned in the literature. Here, we tentatively attribute peak 3′ to the oxidation of the in situ formed W–H* intermediates and/or nascent H_2_ species.^63^ Upon phosphorus doping, the current associated with peak 1′ decreases progressively with increasing P content, consistent with the suppressed proton intercalation inferred from the diminished cathodic peak 1. In contrast, the current of peak 3′ initially increases for the 2% P-OV-WO_3_ catalyst and then decreases at higher P-doping levels, which is likely related to significantly reduced proton intercalation at elevated phosphorus contents.

To further probe hydrogen intercalation dynamics under more kinetically relevant conditions, CV at a higher scan rate (100 mV·s^–1^) was performed with varying cathodic potential limits (Figure S6 in Supporting Information Section S3.6). For C-WO_3_, the peak 1′ current increases, and its peak position shifts positively as more negative cathodic cutoff potentials are applied, indicating progressively increased proton intercalation and enhanced interactions with the WO_3_ matrix. In contrast, for the P-doped samples, the increase in peak 1′ current is markedly attenuated, and the peak position remains largely unchanged with increasing negative cutoff potentials. Collectively, these results demonstrate that increasing the phosphorus content not only suppresses proton intercalation but also modifies W–H* binding characteristics and its reactivity toward H_2_ evolution.

### Relationship between Proton Intercalation and HER Revealed by Rotating Ring-Disk Electrode (RRDE)

In our previous work, we discovered that the “apparent” HER onset potentials of WO_3_-based catalysts derived from CV were much more negative than their true HER onset because the measured cathodic current includes contributions from both proton intercalation and HER, which mask the initial HER signal.^41^ Consequently, it is difficult to quantitively correlate proton intercalation with HER activity using CV alone. To overcome this limitation, we employed a rotating ring-disk electrode (RRDE) configuration to decouple the HER current from the proton intercalation current. Specifically, the catalyst was deposited only onto the glassy carbon (GC) disc electrode (Supporting Information Section S2.1). The potential of the disc electrode was scanned from 1.0 to −0.50 V vs RHE at a scan rate of 10 mV·s^–1^ and 1600 rpm rotation, while the platinum (Pt) ring electrode maintained a constant potential of 0.89 V vs RHE. The hydrogen evolution reaction (HER) current, as reflected by the hydrogen oxidation reaction (HOR) current on the ring electrode, was separated from the total current measured on the disk electrode, which included both HER and proton intercalation currents. The “true” onset HER potential was defined as the potential at which the ring current density reached 1 μA/cm^2^. We assume that the trend of the HOR kinetics measured on the ring electrode represents the trend of the HER kinetics on the disk electrode, where the C-WO_3_ and P-OV-WO_3_ catalysts were deposited.^41^ As shown in Figure 4a, for C-WO_3_, the onset HER potential is ∼−0.06 vs RHE, much more positive than that identified from CV measurements (−0.35 V vs RHE). The onset potential for HER negatively shifted to −0.10 V, −0.15 V, and −0.175 V for the 2.0, 5.0, and 7.5% P-OV-WO_3_ catalysts, respectively. Beyond the HER onset potential of each catalyst, the current density on the ring electrode increases exponentially with increasingly negative disk potentials. Tafel plots constructed from the ring current densities provide insight into the HER kinetics, yielding a Tafel slope of 125 mV dec^–1^ for C-WO_3_ (Figure 4b) and significantly higher slopes of 183, 343, and 355 mV dec^–1^ for the 2.0, 5.0, and 7.5% P-OV-WO_3_ catalysts, respectively. The progressively increased Tafel slopes with phosphorus doping indicate increasingly sluggish HER kinetics. Together with the negatively shifted HER onset potentials, these trends confirm that higher phosphorus doping progressively inhibits HER kinetics in P-OV-WO_3_, consistent with suppressed proton intercalation and delayed W–H* formation. It is worth noting that the HER suppression on P-OV-WO_3_ remains stable over time. No discernible changes in surface chemistry or bulk crystal structure were observed after 5 h of electrolysis (Supporting Information Section S3.7 and Figures S7 and S8).

**Figure 4:**
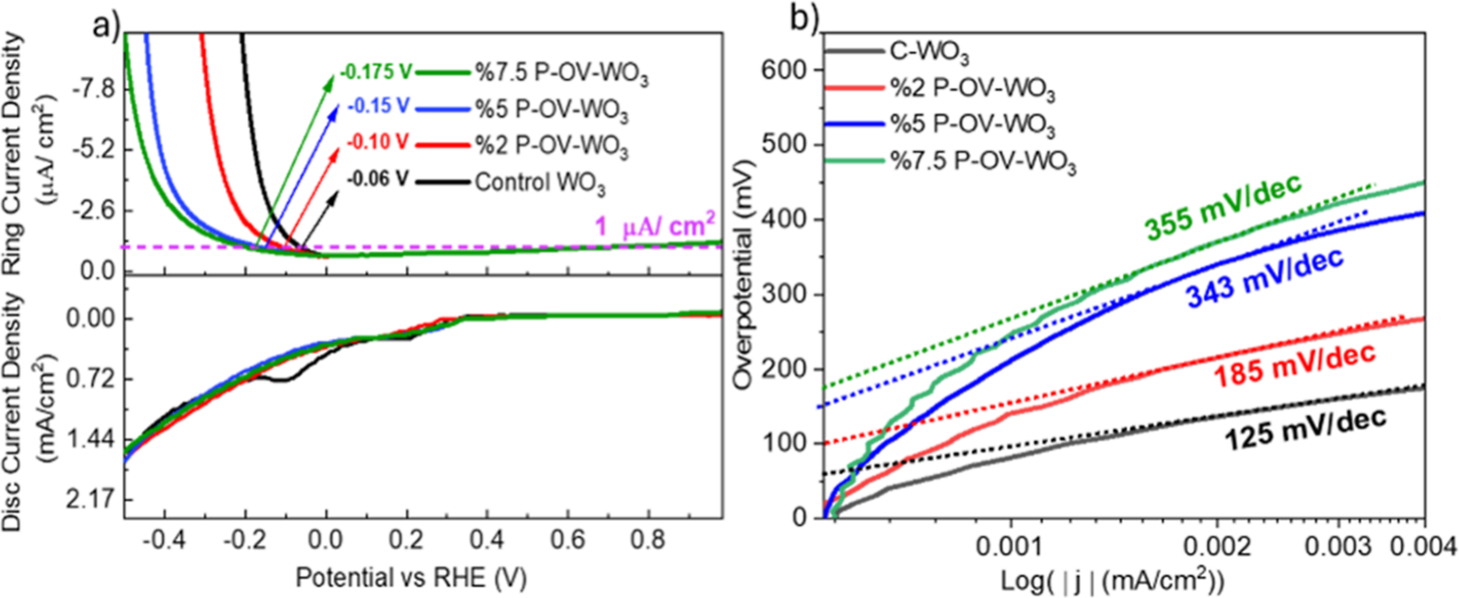
(a) Rotating ring-disk electrode (RRDE) voltammograms of C-WO_3_ and P-OV- WO_3_ of different doping levels in an Ar-saturated pH 2 H_2_SO_4_ electrolyte at 1600 rpm. The negative-going polarization curve on the disk electrode is collected at a 10 mV s^–1^ scan rate at room temperature while the ring potential is held at 0.89 V versus RHE. (b) Corresponding Tafel slopes of C-WO_3_ and P-OV-WO_3_ samples derived from the ring electrode.

### Electrochemical Impedance Spectroscopy (EIS) Study and Quantification of Hydrogen Bond Dissociation Free Energy (H-BDFE)

To elucidate the origin of the observed HER suppression on P-OV-WO_3_ catalysts, a systematic impedance spectroscopy (EIS) study was performed over a carefully selected potential window. The EIS measurements were initiated at the HER onset potential of C-WO_3_, as identified in the RRDE study (−0.06 V RHE, Figure 4a), which is sufficiently negative to ensure that proton intercalation has already occurred for all catalysts. Starting from this reference point, the cathodic potential was progressively increased in 0.05 V increments until reaching −0.36 V vs RHE, where pronounced HER activity becomes evident (Figure 4a). This potential range was deliberately selected to span the transition from the onset of HER for C-WO_3_ and the pre-HER regime for the P-doped catalysts to the onset of the HER-dominated regime. Beyond this regime, significant H_2_ bubble formation occurs at the disk electrode. This window therefore enables detailed interrogation of the charge-transfer and H* adsorption processes associated with the formation of W–H*, corresponding to the Volmer step in the HER. Figure 5a shows the Nyquist plots for all catalysts recorded at −0.36 V vs RHE; Nyquist plots collected at other applied potentials are provided in the Supporting Information Section S3.8 and Figures S9a–d. The Nyquist plots show two arcs representing two electrochemical processes with different time constants. The arc-shaped (non-ideal semicircular) Nyquist plots are typically attributed to surface roughness and vertically porous structures arising from the disordered stacking of nanomaterials on the electrode.^63^ According to previous studies, the impedance response in the high-frequency region is primarily associated with electronic conduction within the catalyst inner layer and interfacial contacts, whereas the low-frequency response originates from the charge-transfer processes occurring at the electrolyte–catalyst interface.^64,65^ To quantitatively resolve these contributions, the EIS data were analyzed using the Armstrong–Henderson double-parallel equivalent circuit model, which has been widely applied to hydrogen-involved electrochemical systems.^63,66–68^

**Figure 5:**
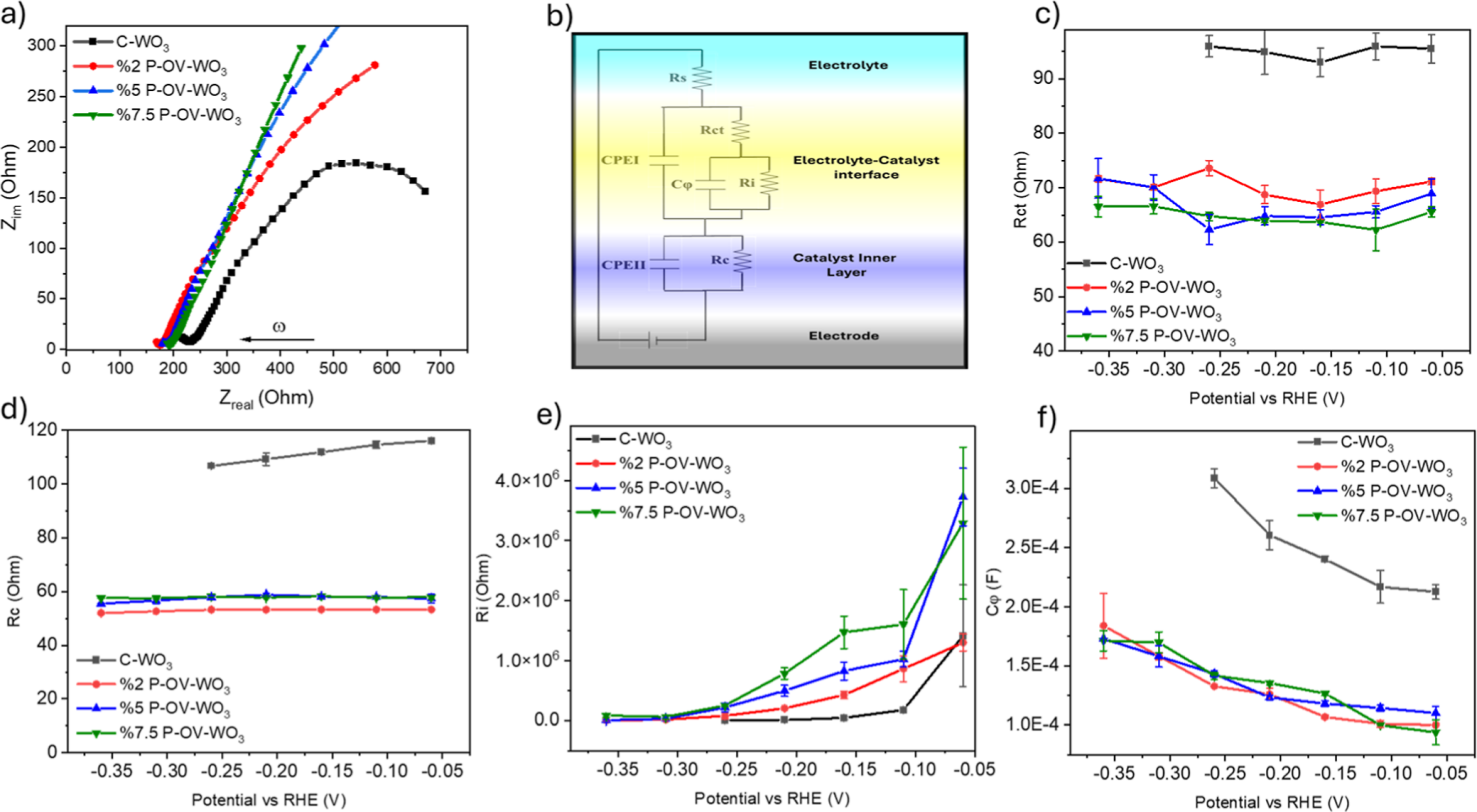
(a) Nyquist plots of all catalysts at −0.36 V vs RHE, (b) Armstrong–Henderson equivalent circuit model. The result of simulated data versus applied constant potentials: (c) *R*ct, (d) *R*c, (e) *R*_i_, and (f) Cφ.

As illustrated in Figure 5b, the equivalent circuit consists of an uncompensated resistance (*R*_s_) in series with two electrochemical subcircuits. The first subcircuit represents the electrolyte–catalyst interfacial processes (yellow region in Figure 5b), which are directly associated with hydrogen adsorption on catalytically active sites, e.g., W sites in WO_3_-based catalysts.^63,66–68^ This interfacial response is described by a charge-transfer resistance (*R*_ct_) in parallel with a constant phase element (CPEI), where *R*_ct_ reflects the interfacial electron-transfer resistance and CPEI accounts for the non-ideal double-layer capacitance arising from surface roughness and interfacial heterogeneity. In series with this interfacial charge-transfer branch, an additional adsorption-related subcircuit is included consisting of an adsorption resistance (*R*_i_) and a pseudocapacitive element (Cφ). *R*_i_ is related to the superficial mass transfer resistance of H* adsorption onto catalytic sites.^69,70^ Cφ reflects the pseudo-capacitance associated with the formation of surface-bound hydrogen intermediates (H*) adsorbed on catalytic sites and is commonly interpreted as being proportional to the surface coverage of adsorbed H* species. Accordingly, an increase in Cφ indicates a higher population of adsorbed hydrogen intermediates, which is often correlated with more facile Volmer kinetics and a greater likelihood of the subsequent Heyrovsky step to H_2_.

The second subcircuit (purple region), composed of a resistance (*R*_c_) and a constant phase element (CPEII), represents the inner layer of the electrode material. Here, the resistance (*R*_c_) signifies the electron transport resistance in the catalyst inner layer, while CPEII accounts for the non-ideal double-layer capacitance arising from surface heterogeneity.

Figure 5c shows the variation of the charge-transfer resistance (*R*_ct_) as a function of applied cathodic potential for all catalysts. The control WO_3_ (C-WO_3_) exhibits relatively constant *R*_ct_ values in the range ∼90–95 Ω across the investigated negative potential window. Over the same potential range, the P-OV-WO_3_ catalysts display slightly lower *R*_ct_ values, with an approximately 20 Ω decrease and only minor variation among different doping levels. Although modest, this reduction in *R*_ct_ indicates that phosphorus doping slightly enhances the intrinsic charge-transfer properties of the WO_3_ framework, suggesting that the suppressed HER activity observed for P-OV-WO_3_ is unlikely to originate from changes in interfacial charge-transfer kinetics.

Figure 5d shows the catalyst inner electron transport resistance (*R*_c_) as a function of applied cathodic potential for P-OV-WO_3_ catalysts and C-WO_3_. At −0.06 V vs RHE, C-WO_3_ exhibits an Rc of approximately 116.2 Ω, whereas the 2%, 5%, and 7.5% P-OV-WO_3_ catalysts display substantially lower *R*_c_ values (∼52–60 Ω). With increasing cathodic polarization, the Rc values for the P-OV-WO_3_ catalysts remain nearly constant. While the *R*_c_ for C-WO_3_ decreases slightly up to −0.26 V, data at more negative potentials are not shown due to pronounced H_2_ bubble formation (Figure 3a). This slight decrease in *R*_c_ for C-WO_3_ is consistent with progressive proton intercalation into WO_3_. Although a semiconductor-to-metal transition occurs upon the initial proton intercalation at ∼0.3 V versus RHE, further proton intercalation at more negative potentials is associated with continued improvement in electrical conductivity. In contrast, the consistently lower *R*_c_ values of the P-OV-WO_3_ catalysts indicate intrinsically more efficient electron transport, as supported by Mott–Schottky analysis (Figure S5). The lower *R*_c_ or increased conductivity of the P-OV-WO_3_ catalysts is also consistent with their observed lower *R*_ct_ values (Figure 5c), reflecting faster interfacial charge-transfer kinetics. Accordingly, electron transport limitations are unlikely to account for the suppressed HER activity in P-OV-WO_3_ either, which is instead attributed to differences in hydrogen intermediates (W–H*), as discussed below.

Figure 5e,f collectively elucidates the impact of phosphorus doping on hydrogen intermediate (W–H*) formation on WO_3_. At −0.06 V vs RHE, all catalysts exhibit high W–H* adsorption resistance (*R*_i_ > 1.0 × 10^6^ Ω), with C-WO_3_ showing the lowest *R*_i_ and 7.5% P-OV-WO_3_ the highest (Figure 5e). With increasing cathodic polarization, the Ri decreases for all samples. However, C-WO_3_ exhibits a rapid decline at relatively mild potential, indicating facile hydrogen adsorption and early HER onset. In contrast, all of the P-OV-WO_3_ catalysts retain significantly higher *R*_i_ values until more negative potentials are reached, with pronounced decreases occurring only at ∼ −0.21 V for 2% P-OV-WO_3_ and ∼ −0.31 V for 5% and 7.5% P-OV-WO_3_, reflecting delayed W–H* formation. Consistently, the hydrogen adsorption pseudocapacitance (Cφ) of C-WO_3_ increases nearly linearly from ∼0.21 to ∼0.31 mF with increasing cathodic potential, indicative of steadily increasing surface hydrogen coverage (Figure 5f). In contrast, all P-OV-WO_3_ catalysts exhibit substantially lower initial Cφ values (∼0.12 mF) and only modest increases to ∼0.18 mF, with minimal dependence on the doping level. Together, the persistently higher *R*_i_ and decreased Cφ values indicate that phosphorus doping weakens hydrogen adsorption on WO_3_ by modifying the surface electronic structure, thereby delaying W–H* formation and reducing surface H* coverage.

We further quantified the hydrogen bond dissociation free energy (H-BDFE) of the two catalysts (Supporting Information Section S3.9 and Figure S10). Following the reported methodology,^71^ the H-BDFE values were estimated to be 54.9 ± 1.22 kcal/mol for P-OV-WO_3_ and 57.2 ± 0.78 kcal/mol for C-WO_3_. The lower H-BDFE of P-OV-WO_3_ indicates a thermodynamically weaker H surface interaction, which facilitates hydrogen atom transfer and, in principle, could favor H_2_ formation. However, the HER activity of catalysts is not governed solely by H-BDFE but is strongly dependent on H* surface coverage. In our system, P-OV-WO_3_ exhibits reduced H* coverage, as described above, limits the availability of reactive intermediates, and kinetically suppresses H_2_ formation despite the lower H-BDFE.

### Computational Electronic Structure Analysis

Our computational analysis focuses on the changes in the electronic structure of bulk C-WO_3_ as well as P-OV-WO_3_ due to the first hydrogen (H) intercalation. Several computational studies have investigated hydrogen intercalation into bulk or surface C-WO_3_.^58,60^ Numerous computational studies have also investigated the effect of various doped-elements on the electronic structure of C-WO_3_ [e.g., Pd,^72,73^ Pt,^74^ Au,^74^ and Ti^75,76^ ]. However, P-doping and its effect on bulk H-intercalation have not received any attention to the best of our knowledge. Here, to complement our experimental observations, we performed DFT calculations to elucidate the effects of phosphorus (P) doping on the structural and electronic properties of WO_3_. The details of our calculations can be found in the Supporting Information (Supporting Information Section S4).

Briefly, we used PBE-D3 DFT calculations to investigate the changes in the electronic structure of monoclinic C-WO_3_ (8 W atoms and 24 O atoms) and its P-doped counterpart with an O-vacancy (P-OV-WO_3_) consistent with the experimentally characterized structures. This level of P-doping corresponds to a P-doping level of 12.5%. The choice of our computational method is explained in Supporting Information Section S4. The P-OV-WO_3_ structure is generated by substitutional doping of one P atom next to the O-vacancy created in the C-WO_3_ cell. Even for a simulation cell of this size, the number of H-intercalation calculations can ramp up very rapidly (_24_C_n_ and _23_C_n_ for C-WO_3_ and P-OV-WO_3_, respectively, considering all O-sites, where n is the number of intercalated H atoms). The first H-intercalation corresponding to the stoichiometry of H_0.125_WO_3_ or H_0.125_P-OV-WO_3_ is considered here. Considering all of the possible H-intercalation sites, even this first H-intercalation in C-WO_3_ and P-OV-WO_3_ would lead to ∼50 DFT calculations. Although a higher level of H-intercalation can be achieved experimentally,^57,58^ explicit treatment of multiple intercalations rapidly becomes computationally prohibitive (e. g. ∼20000 configurations for H_0.500_WO_3_). Importantly, the first H-intercalation captures valuable insights into the changes in the electronic structures of C-WO_3_ and P-OV-WO_3_ by hydrogen incorporation and is sufficient to rationalize the experimental trends. Accordingly, we limit our computational analysis to the first H-intercalation step, while higher order effects will be studied in the future. In addition, details of the DFT methodology for modeling proton intercalation, including justification for the adsorption energy approach, are provided in Supporting Information Section S4.

First, the structures of C-WO_3_ and P-OV-WO_3_ before H-intercalation were geometry optimized (Figure S11). These optimized structures were then used to understand the overall changes in the electronic structure of C-WO_3_ due to P-doping, as well as H-intercalation.

Figure 6 shows the partial density of state (PDOS) of C-WO_3_ with the d-band center of W and the p-band center of O identified using a vertical dashed line. As expected,^57,58^ C-WO_3_ shows a semiconductor character clearly shown by the bandgap observed near the Fermi energy level. The first H-intercalation results in an overall or average negative shift of both O p-band and d-band centers of the p-band of the W d-band accompanied by a switch from semiconductor to degenerate semiconductor or metal character, in agreement with experimental Mott–Schottky analysis presented in Figure S5. This observation also matches the conclusions from Miu et al.,^57,58^ who also noted a similar change in the WO_3_ electronic structure after H-intercalation. Notably, even in the absence of hydrogen intercalation, P-doping induces an overall negative shift of both the W d-band and the O p-band, resulting in a degenerate semiconductor character and increased electronic conductivity (Figure 7). This trend is consistent with our experimental observations from the Mott–Schottky analysis described earlier. The first H-intercalation in the P-OV-WO_3_ structure shifts the O p-band center further away from the Fermi energy level similar to C-WO_3_, whereas the W d-band center in P-OV-WO_3_ shifts positively, opposite to the trend observed in C-WO_3_.

**Figure 6:**
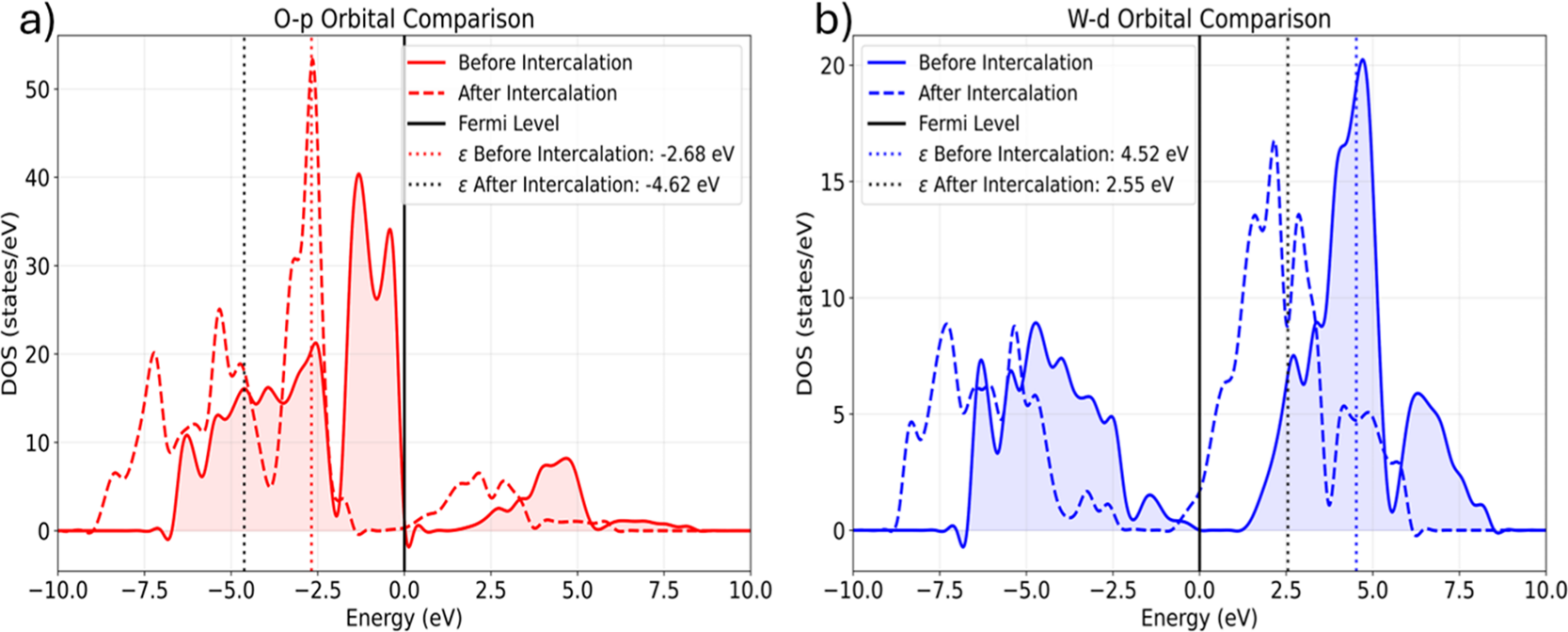
Projected density of states (PDOS) of (a) O p orbitals and (b) W d orbitals for WO_3_ before and after hydrogen intercalation. Solid lines represent the pristine structure, while dashed lines correspond to the H-intercalated structure. The vertical black line denotes the Fermi level (set to 0 eV). Hydrogen intercalation induces a pronounced shift in both the O *p*-band and W *d*-band centers relative to the Fermi level, reflecting significant modulation of the electronic structure upon proton intercalation.

**Figure 7:**
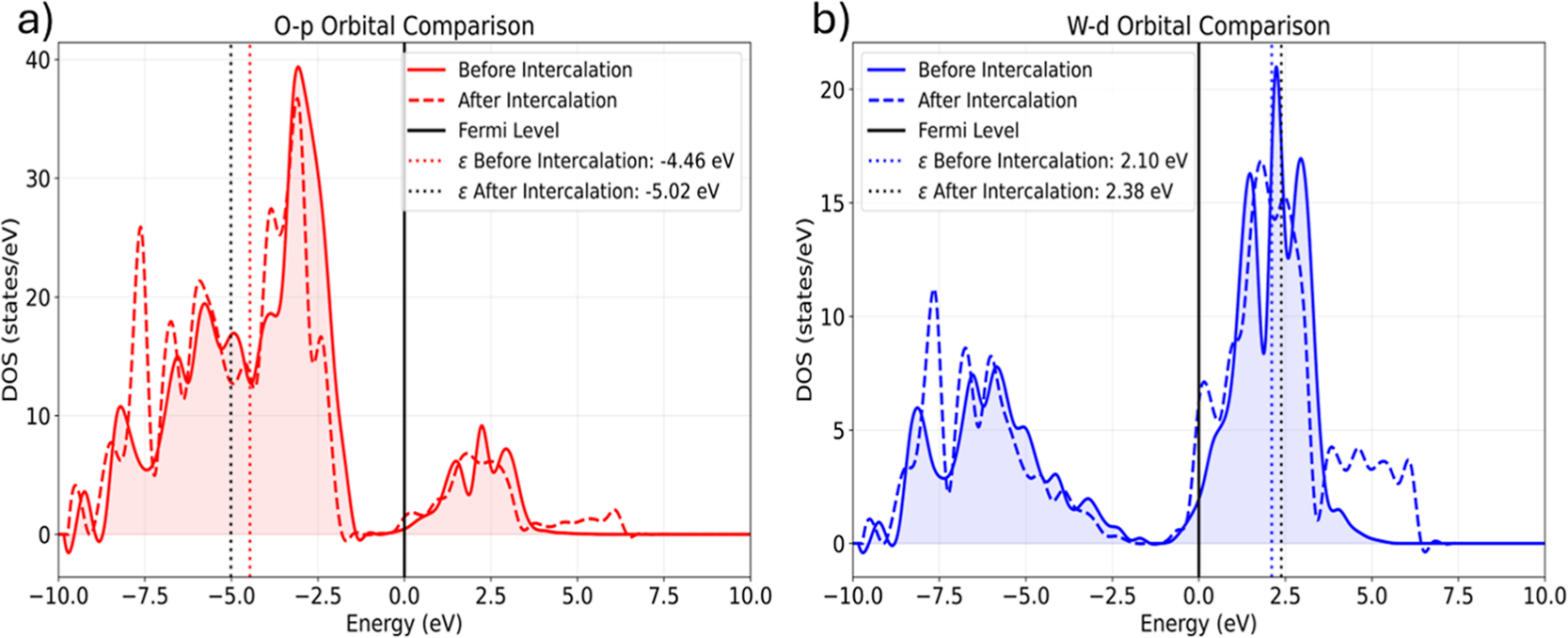
Projected density of states (PDOS) of (a) O p orbitals and (b) W d orbitals for WO_3_ before and after hydrogen intercalation. Solid lines correspond to the pristine structure, while dashed lines represent the H-intercalated structure. The Fermi level is set to 0 eV (vertical black line). Hydrogen intercalation induces a downshift of the O *p*-band center (from −4.46 to −5.02 eV) and an upshift of the W *d*-band center (from 2.10 to 2.38 eV).

We note that PBE functionals underestimate band gaps in oxide materials; however, benchmark HSE06 calculations confirm that the qualitative electronic structure trends reported here are preserved (Supporting Information Section S5 and Figure S16).

Next, the first H-intercalation was systematically examined at all possible intercalation sites in both C-WO_3_ and P-OV-WO_3_ structures, and the calculated hydrogen binding energies for all examined sites are summarized in Supporting Information Section S4 and Figures S12 and S13. The highest H-binding energies on the O-sites are −0.46 eV for C-WO_3_ and −0.93 eV for P-OV-WO_3_. However, the average binding energy on the O-sites reduces to −0.25 eV for P-OV-WO_3_, which is much lower than that for C-WO_3_ (−0.39 eV). The H-binding energy values for C-WO_3_ match well with the analysis of Miu et al. using DFT calculations.^57,58^ The results clearly show the difference in H-intercalation behavior with P-OV-WO_3_ compared to C-WO_3_. The introduction of P along with O-vacancy not only changes the material toward a more metallic electronic character but also changes the H-intercalation behavior. For the first H-intercalation in C-WO_3_, the binding energies on different O-sites show a standard deviation of 0.03 eV. This means that the first H-intercalation to any of the O-sites is energetically similar owing to slight differences in H-binding energies across different O-sites. In P-OV-WO_3_, the H-binding energies show a much larger standard deviation of 0.25 eV. Unlike C-WO_3_, where all sites show binding energies within 0.39 ± 0.03 eV, more than half of the O-sites (13 out of possible 23) in P-OV-WO_3_ show a H-binding energy of −0.22 eV or less. Six O-sites show H-binding energy within −0.39 ± 0.3 eV with three O-sites showing binding energies of −0.6 eV or higher. Hence, despite some of the sites showing more favorable H-binding energies leading to higher H-intercalation, the overall kinetics of the first H-intercalation in P-OV-WO_3_ should be more sluggish as compared to C-WO_3_.

To further analyze the changes in the electronic structure, we calculated the Bader charges in both C-WO_3_ and P-OV-WO_3_ structures. The key observations from Bader charge analysis are as follows: In P-OV-WO_3_, the average partial charge for O atoms became more negative (−1.04) as compared to the average partial charge of O atoms in C-WO_3_ (−0.98). In fact, the O atoms geometrically closest to the P atom (Supporting Information Section S4 and Figure S11) show the lowest (more negative) partial charges, as listed in Table 1. This change in partial charge is expected as the vacancy in the O atom leads to excess electrons that get dispersed due to the metallic nature of P-OV-WO3, leading to more negative partial charges on the O atoms. Out of these four O-sites, three sites, namely, O1, O15, and O19, are bonded strongly to W atoms, resembling the C-WO_3_ lattice. O17, on the other hand, has a much weaker/no W–O bond, as evidenced by a longer W–O distance, indicating weakened W–O coordination. While PXRD (Figure 1b) probes the average structure rather than site-specific bonds, the observed low-angle 2θ shifts and peak broadening with P-doping are consistent with an overall increase in interplanar spacing and bond-length/angle distortions that support such local coordination weakening. While all of these sites carry a higher negative charge, the lack of strong hybridization with W d-states of O17 likely hinders the delocalization of the electron introduced by H-intercalation. Consequently, despite the Coulombic attraction for the proton, the overall binding affinity for O17 is reduced as compared to O1, O15, and O19 as well as the average H-binding energy for the O-sites (0.39 eV) in C-WO_3_ that allows efficient charge transfer to the W d-band. Distinct from the charge-driven binding observed at sites O1 and O19, site O9 exhibits a high H-binding affinity (−0.64 eV) without possessing an exceptionally negative Bader charge. We attribute this strong binding to the relief of local lattice strain rather than electrostatic attraction. The introduction of P-doping and oxygen vacancies also induces significant local distortion in the tungsten-oxide framework. Upon H-intercalation at O9, we observe a massive reconstruction of the local coordination environment: the O–W–O bond angle compresses significantly from 98.7° to 83.9°. This sharp angular change (Δ ≈ 15°) suggests that the preintercalation structure is highly strained in this region. The insertion of a hydrogen atom facilitates geometric relaxation, effectively releasing this stored elastic strain energy. Thus, the favorable binding energy at O9 is primarily mechanical in origin, driven by the stabilization of the distorted lattice geometry.

**Table 1:** Properties of O-Atoms Structurally Closest to the P-Atom in P-OV-WO_3_ ^a^

O-site	partial charge	Δ partial charge	W–O distance (Å)	H-binding energy (eV)
O1	–1.24	0.26	1.85	–0.64
O15	–1.40	0.42	2.02	–0.46
O17	–1.41	0.43	3.19	–0.34
O19	–1.23	0.25	1.87	–0.78

a See Supporting Information Figure S11 for visualization of the O-sites.

We also calculated the H-equilibrium potential for the first H-intercalation by using the computational hydrogen electrode model for all the H-binding sites (see Supporting Information Figures S14 and S15).^77^ Similar to the trends in H-binding energies, the introduction of P-doping significantly changes the equilibrium potential for H-intercalation. As the equilibrium potential for first H-intercalation is inversely proportional to the H-binding energy, the average equilibrium potential reduces from 0.39 to 0.25 V upon P-doping. This trend qualitatively agrees with the experimental observation for the first proton intercalation peak from 0.17 to 0.13 V upon 7.5% of P-doping (See Figure 3b). The quantitative disagreement between the experiments and simulations for the first H-intercalation was also noted by Miu et al. in their investigation of H-intercalation in WO_3_.^57,58^

In summary, our DFT calculations indicate that P-doping along with an O-vacancy changes the semiconductor nature of C-WO_3_. The P-doping along with the presence of OV leads to a multitude of rather complex effects that could affect the H-intercalation behavior including P–O bonding, proximity of O-sites to OV, and straining of the W–O–W lattice due to the presence of P-doping and OV (supported by PXRD results). The substitution of P does significantly change the first H-intercalation behavior of O-sites. While some O-sites show higher H-binding energy, more than half of the O-sites show much lower H-binding energy compared to those of the O-sites in C-WO_3_. This leads to overall sluggish kinetics for the first H-intercalation, as observed in the experiments, and a reduction in the average binding energy for H. The average equilibrium potential for the first H-intercalation also reduces corroborating experimental observations. Considering the interesting findings shown here, a more detailed computational investigation into further H-intercalation steps, reaction on the catalyst surface, and other doping candidates will be undertaken as a part of the future work.

## Conclusion

In this work, we elucidate a fundamentally different pathway for suppressing the hydrogen evolution reaction in tungsten oxide-based electrocatalysts through metalloid phosphorus doping. Contrary to conventional oxygen-vacancy-rich WO_3_ systems, where increased conductivity and enhanced H* adsorption promote HER, P-OV-WO_3_ exhibits simultaneously improved electronic conductivity and strongly suppressed HER activity. Cyclic voltammetry and rotating ring-disk electrode measurements reveal that phosphorus doping progressively inhibits proton intercalation, leading to negatively shifted HER onset potentials and substantially increased Tafel slopes. Electrochemical impedance spectroscopy, Mott–Schottky analysis, and H-BDFE measurement further show that, despite faster interfacial charge transfer, improved inner electronic conductivity, and decreased H-BDFE, phosphorus doping significantly increases the interfacial resistance associated with H* adsorption, leading to reduced surface coverage of W–H* intermediates and thereby suppressing HER kinetics. DFT calculations further show that phosphorus doping introduces substantial site-to-site heterogeneity in hydrogen binding across oxygen sites, broadening the distribution of H* adsorption energies. As a result, although the average W d-band center shifts closer to the Fermi level, an effect that would typically favor stronger hydrogen adsorption, many oxygen-coordinated sites bind H* too weakly to support efficient proton intercalation, thereby limiting the surface H* population. Together, these results establish that HER suppression can also be achieved through regulating local hydrogen adsorption rather than limiting conductivity and the charge transfer process and tuning the d band centers of the catalytic sites, offering a broadly applicable novel strategy for designing selective electrocatalysts for proton-coupled electroreduction reactions.

## Supplementary Material



## Notes

Detailed catalyst characterization; catalyst ink preparation and deposition procedures; representative SEM images of the H_2_WO_4_ precursor, C-WO_3_, and P-OV-WO_3_ catalysts; representative EDS spectra and elemental mapping of P-OV-WO_3_; expanded PXRD spectra of P-OV-WO_3_ catalysts; XPS quantification tables; Mott–Schottky plots and CV results for different catalysts at various cathodic potential cutoffs obtained at higher scan rates; Nyquist plots, equivalent-circuit fitting parameters, and capacitance fitting results for all catalysts at different potentials; and computational details including methods and results for DFT-optimized structures of C-WO_3_ and P-OV-WO_3_, hydrogen binding energies, and hydrogen intercalation equilibrium potentials at all oxygen sites of both catalyst models (PDF)
