# Supplementary material for: Mechanistic Insights into the Suppression of Proton Intercalation and the Hydrogen Evolution Reaction through Phosphorus Doping in Tungsten Oxide
Source: ACS Electrochem. 2026 May 6;2(6):1383–96. doi: 10.1021/acselectrochem.6c00059 (PMC13244369; doi:10.1021/acselectrochem.6c00059)
Supplement: Supplementary file 1 [file ec6c00059_si_001.pdf]

Supporting Information for

Mechanistic Insights into the Suppression of Proton Intercalation and Hydrogen Evolution Reaction through Phosphorus Doping in Tungsten Oxide

Oguz Kaan Kucukosman [a], Hengfei Gu [b], Xueyuan Zhang [c], Jacob Smith [d], Zhiyuan Zhang [a], Elizabeth Desmet[e], Yiguang Ju [e], Eric Garfunkel [b], Miaofang Chi [d, e], Aditya Dilip Lele [f *], Huixin He [a*]

[a] *Department of Chemistry, Rutgers, the State University of New Jersey, Newark, NJ 07102*

[b] *Department of Chemistry and Chemical Biology, Rutgers University, New Brunswick, New Jersey 08901, United States*

[c] *Gamry Instruments, Inc., Warminster, PA, 18974, United State*

[d] *Center for Nanophase Materials Sciences, Oak Ridge National Laboratory, Oak Ridge, Tennessee 37831, United States*

[d] *Department of Mechanical and Aerospace Engineering, Princeton University, Princeton, New Jersey 08544, United States*

[f] *Thomas Lord Department of Mechanical Engineering & Materials Science, Duke University, Durham, North Carolina 27708, United States*

[g] *Department of Mechanical Engineering, Rowan University, Glassboro, New Jersey 08028, United States*

Corresponding Author: *huixinhe@newark.rutgers.edu and lele@rowan.edu*

Table of Contents

S1. Material Characterization & Electrochemical Measurement. S3

S2. Preparation of catalyst ink, deposition of catalyst onto electrodes, and Pt counter electrode validation S3

S2.1 Preparation of catalyst ink, deposition of catalyst onto electrodes. S3

S2.2 Pt Counter-Electrode Validation Using RRDE Measurements S4

S3. Structural Features of P-OV-WO_3 and C-WO_3 Catalysts S5

S3.1 Morphological Characterization of C–WO_3 and P-OV-WO_3 by SEM S5

S3.2 P doping and P doping distribution in P-OV-WO_3 by Energy-Dispersive X-ray Spectroscopy and Energy-dispersive X-ray mapping S6

S3.3 Detailed PXRD Analysis of Structural Disorder and Partial Amorphization in P-Doped WO_3 S7

S3.4 Detailed XPS Peak Deconvolution and Chemical State Analysis S8

S3.5 Mott–Schottky Analysis of Charge Carrier Density in C–WO_3 and P-OV-WO_3 Catalysts S9

S3.6 Behavior of Proton Intercalation and deintercalation at fast scanning rate of (100 $mV{\cdot}s^{-1}$) S10

S3.7 Stability test S11

S3.8 Electrochemical Impedance Spectroscopy (EIS) Studies: Nyquist Plots and Equivalent Circuit Fitting S14

S3.9 Hydrogen bond dissociation free energy (H-BDFE) measurements S18

S4 Computational Details S20

S5. Benchmark discussion of PBE-D3 vs HSE06 S28

S1. Material Characterization & Electrochemical Measurement.

The morphology of the synthesized P-OV-WO_3 and C-WO_3 catalysts were examined using field-emission scanning electron microscopy (FE-SEM; Hitachi S-4800 and JSM-7900F, JEOL, Japan) operated at an accelerating voltage of 15 kV. The crystalline phase identification was performed by X-ray diffraction (XRD) on a Rigaku Miniflex 6G diffractometer using Co Kα radiation (λ = 1.789 Å). Surface chemical composition and electronic states of the catalysts were characterized by X-ray photoelectron spectroscopy (XPS; Thermo K-Alpha, USA) with monochromatic Al Kα radiation (hv = 1486.6 eV). All XPS spectra were calibrated using the C 1s peak at 284.8 eV as an internal standard. For data analysis, Smart background subtraction was applied, and the spectra was fitted with Gaussian/Lorentzian peaks using a minimum deviation curve fitting routine implemented in the Advantage software. The surface composition of each species was quantified from integrated peak areas and the Scofield sensitivity factor provided by the software was applied.

S2. Preparation of catalyst ink, deposition of catalyst onto electrodes, and Pt counter electrode validation

S2.1 Preparation of catalyst ink, deposition of catalyst onto electrodes.

Precise preparation of the catalyst ink involved dispersing 5 mg of the catalyst in a solution of 750 µL deionized water, 250 µL isopropanol, and 5 µL Nafion (5 w/w%, Beantown Chemicals). After 30 minutes of ultrasonic dispersion, 10 µL of the resulting ink was meticulously placed on glassy carbon disk electrode of a Rotated Ring Disc electrode (RRDE, ALS Co. Ltd, Tokyo, Japan). The RRDE itself consisted of a 4 mm diameter glassy-carbon disk surrounded by a Pt ring. The homogeneous deposition process was facilitated through spin coating at 300 RPM, utilizing an RRDE-3A rotator (ALS Co. Ltd, Tokyo, Japan), and continued until the catalyst reached complete

dryness. Noteworthy, all electrochemical measurements were applied via RRDE electrode to increase consistency in Ar saturated electrolyte (5 mM H_2SO_4, pH= 2).

S2.2 Pt Counter-Electrode Validation Using RRDE Measurements

It has been reported that Pt counter electrodes may undergo electrochemically induced oxidative dissolution and redeposition onto the working electrode, especially at high currents and fast scan rates, resulting in artificially enhanced HER activity that does not reflect the intrinsic catalytic performance of the tested catalyst.[1] To address this issue, the Allam group systematically tested different counter electrodes and confirmed that Ti-based counter electrodes remain stable and suitable for reliable HER measurements in acidic media.[2] Although this work investigates HER suppression (rather than enhancement) by P doping in WO_3-based catalysts and the operating currents were relatively low (in the mA range), we nevertheless carried out carefully designed control experiments to rule out possible artifacts from Pt counter electrode dissolution/redeposition. RRDE measurements were first carried out on C-WO_3 catalysts during HER studies using both Pt and Ti counter electrodes. Specifically, three sequential measurements were performed on the same catalyst-coated RRDE electrode: (i) using Ti as the counter electrode, (ii) using Pt as the counter electrode, and (iii) using Ti as the counter electrode with the electrolyte previously used in (ii), to assess potential carryover effects from Pt dissolution. For each condition, 25 linear sweep voltammetry (LSV) scans were collected after an equilibration period of 100 s at open-circuit potential (OCP). The same protocol and analysis were applied to the 5 wt% P-OV-WO_3 catalyst. As shown in **Figure S1**, the nearly identical ring and disk current–potential curves obtained under the three conditions for both C-WO_3 and 5 wt% P-OV-WO_3, indicating negligible Pt counter-electrode dissolution and redeposition and no measurable impact on proton intercalation

or HER behavior under the present experimental conditions. Therefore, all electrochemical characterizations reported in this work were performed using Pt as the counter electrode.

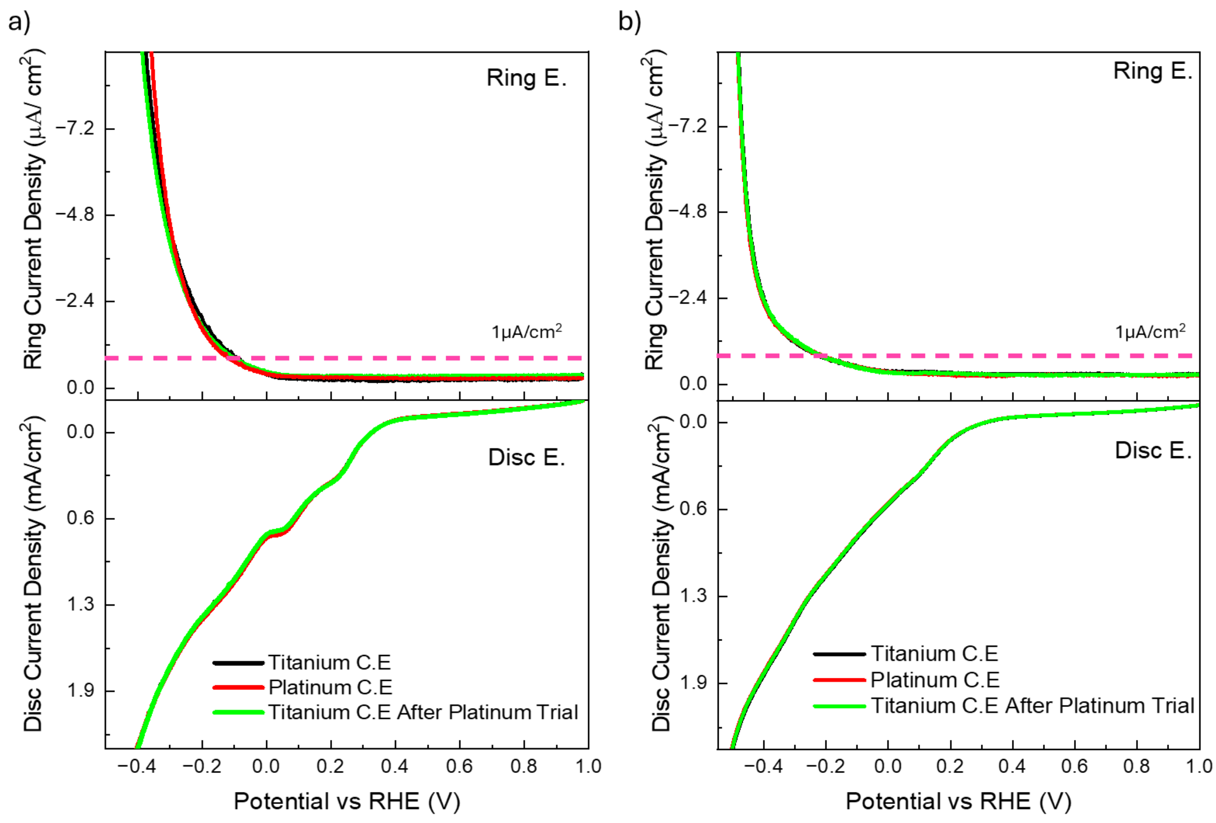

Figure S1. Counter-electrode validation of RRDE-derived HER onset for (a) C-WO_3 catalysts, and (b) %5 P-OV-WO_3. Top panel: Ring current density versus disk potential under Ti counter electrode (black), Pt counter electrode (red), and Ti counter electrode following Pt counter electrode measurements (green). The dashed line indicates the HER onset criterion defined as ring current density = 1 μA.cm^{-2}. Bottom panel: Corresponding disc current density responses. The HER onset potential determined from the ring current response is unchanged across counter electrode configurations.

S3 Structural Features of P-OV-WO_3 and C-WO_3 Catalysts

S3.1 Morphological Characterization of C–WO_3 and P-OV-WO_3 by SEM

SEM images of H_2WO_4 precursor, C-WO_3, %2 and 7.5% (w/w) P-OV-WO_3 nanosheets are in **Fig. S2**. They are square nanosheets with a length of 100~350 nm and thickness of approximately 10

to 30 nm. While the edge of WO_3 was slightly rounded, all the P-doped samples retained similar square nanosheets, consistent with our previous report.[3]

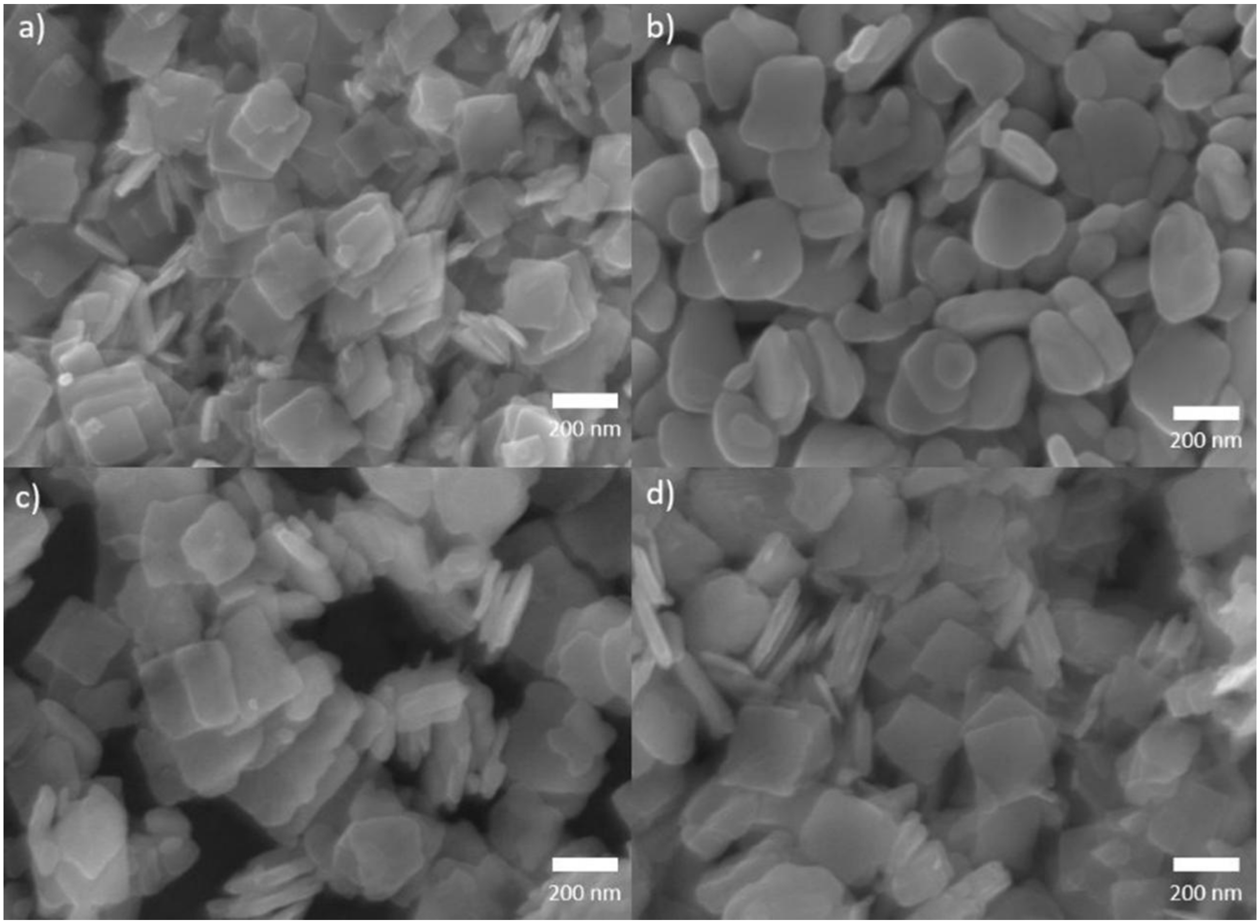

Figure S2. SEM images of H_2WO_4 precursor (a), C-WO_3 (b), %2 P-OV-WO_3, (c) and (d) %7.5 P-OV- WO_3

S3.2 P doping and P doping distribution in P-OV-WO_3 by Energy-Dispersive X-ray Spectroscopy and Energy-dispersive X-ray mapping

As shown in **Figure S3**, P is successfully doped into the WO_3 matrix, with a uniform spatial distribution throughout the catalyst as revealed by elemental mapping.

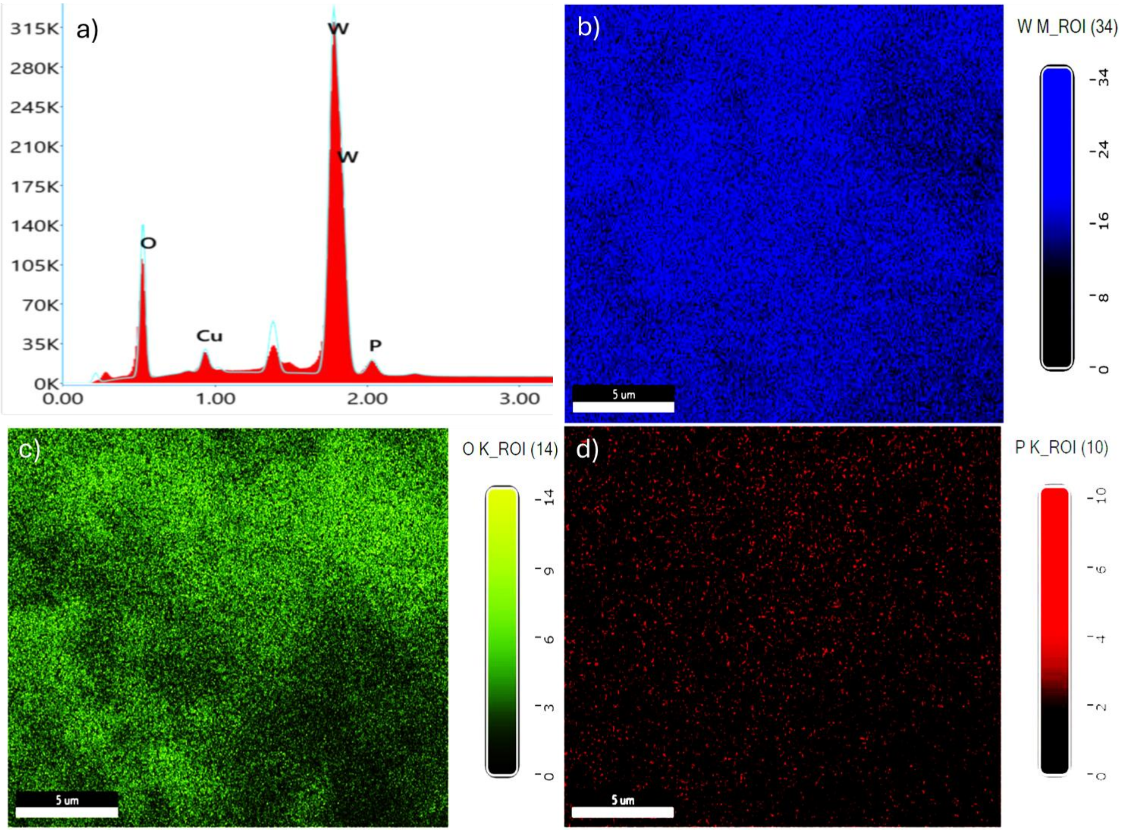

Figure S3. A representative Energy-Dispersive X-ray Spectroscopy (a) and Energy-dispersive X-ray mapping of W (b), O (c) and (d) of %7.5 P-OV-WO_3.

S3.3 Detailed PXRD Analysis of Structural Disorder and Partial Amorphization in P-Doped WO_3

The crystal structures of C-WO_3 and P-OV-WO_3 catalysts were characterized via powder X-ray diffraction (PXRD). The PXRD pattern of C-WO_3 (**Fig.1b**) is a monoclinic crystal structure.[4] Similarly, all P-OV- WO_3 catalysts display monoclinic structural patterns; however, the peaks corresponding to P-OV-WO_3 are significantly broadened and show reduced intensities. The observed peak broadening and reduced diffraction intensity suggests that phosphorus doping induces substantial structural disorder and partial amorphization of WO_3, with higher P doping leading to progressively decreased crystallinity. Closer inspection of specific diffraction planes provides insight into the structural evolution upon P doping. The (002) reflection **(Figure S4a)**

shifts to lower 2θ values with increasing phosphorus content, indicating an expansion of the interplanar spacing along this crystallographic direction. While the (202) reflection remains essentially unchanged, the (022) and (222) reflections of P-OV-WO_3 shifts to lower angles as the phosphorus concentration increases (**Fig. S4b and S4c**), which is consistent with previous works.[3, 5]

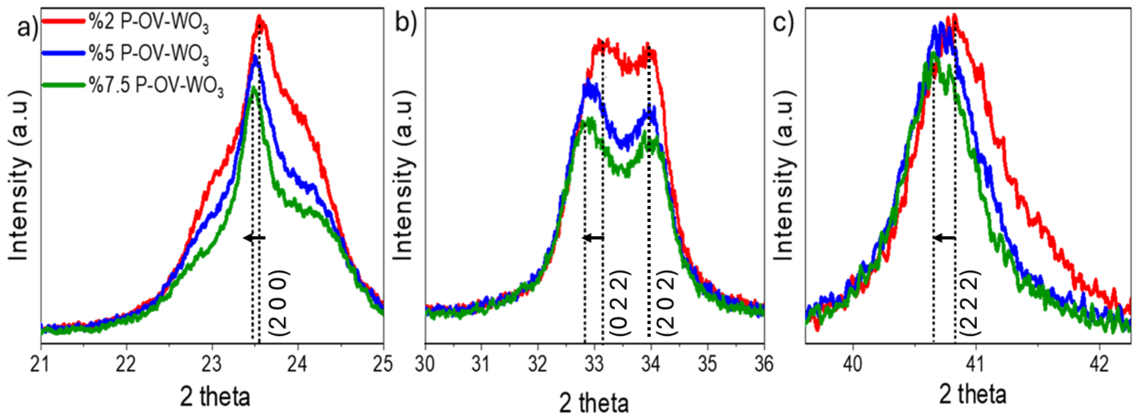

Figure S4. Enlarged PXRD patterns of P–OV–WO_3 catalysts with different phosphorus loadings (2, 5, and 7.5 wt.%) highlighting the shifting of monoclinic WO_3 reflections: (a) (200), (b) (022), and (c) (222).

S3.4 Detailed XPS Peak Deconvolution and Chemical State Analysis

Table S1: XPS quantification of deconvoluted peaks shown in **Figure 2**.

Sample	W(VI) at%	W(V) at%	OL (Lattice Oxide) at%	OH/OL ratio	Doping Phosphorus at%	W(V)/W (total)	OL/(W+P)
C-WO_3	30.8 ± 0.1	N/A	69.2 ± 0.1	0.065 ± 0.005	N/A	N/A	2.25 ± 0.01
% 2 P-OV-WO_3	27.2	2.2	67.2	0.18	3.5	0.07	2.04
% 5 P-OV-WO_3	26.6 ± 0.8	2.65 ± 0.65	66.35 ± 0.45	0.23	4.40 ± 0.3	0.09 ± 0.02	1.97 ± 0.04
% 7.5 P-OV-WO_3	23.9 ± 1.1	3.8 ± 1.1	66.45 ± 0.25	0.275 ± 0.005	5.85 ± 0.25	0.14 ± 0.04	1.98 ± 0.02
at%: Atomic Percentage							

Table S2: Binding Energy of deconvoluted peaks shown in **Figure** 2

Sample	W(VI) B.E. (eV)	W(V) B.E. (eV)	OL (Lattice Oxide) B.E. (eV)	OH (Hydroxide) B.E. (eV)	Doping Phosphorus B.E. (eV)
C-WO_3	35.9	N/A	530.5	531.95 ± 0.5	N/A
% 2 P-OV-WO_3	36.3	35	530.9	532.5	134.2
% 5 P-OV-WO_3	36.4	34.8 ± 0.1	531.0	532.6	134.25 ± 0.5
% 7.5 P-OV-WO_3	36.45 ± 0.5	35.15 ± 0.25	531.1	532.8	134.35 ± 0.5

S3.5 Mott–Schottky Analysis of Charge Carrier Density in C–WO_3 and P-OV-WO_3 Catalysts

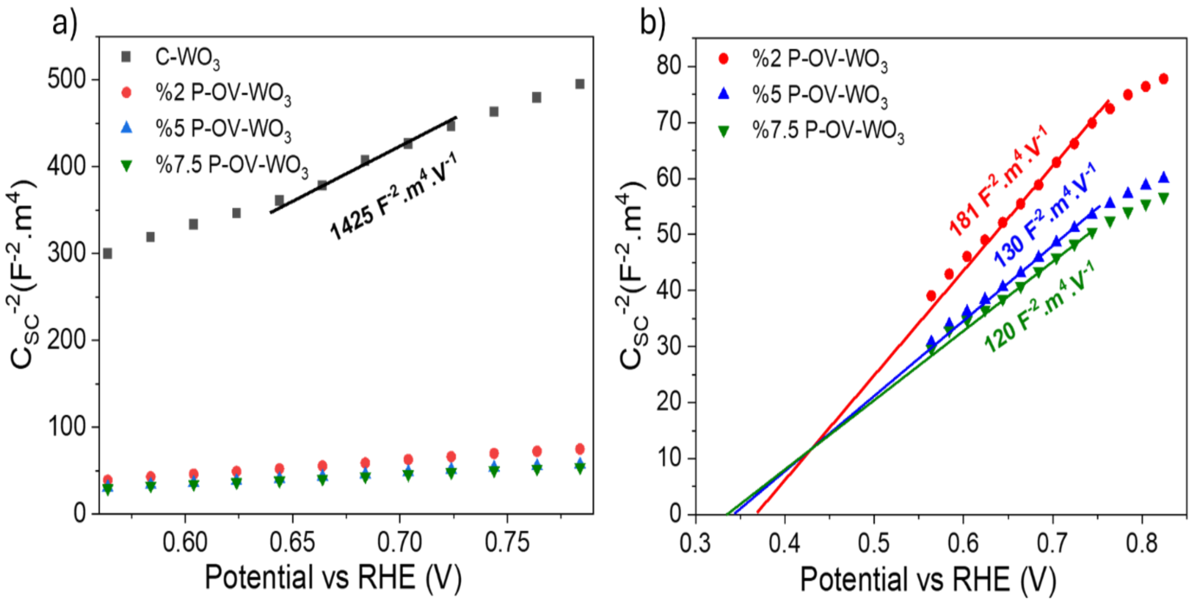

Figure S5. (a) Mott-Schottky plots of the C-WO_3 and P-OV-WO_3 catalysts. (b) Expanded view of panel (a) highlighting the P-OV-WO_3 catalyst.

S3.6 Behavior of Proton Intercalation and deintercalation at fast scanning rate of (100 mV·s^{-1})

To complement the slow-scan CV analysis and explore the dynamics of hydrogen uptake under faster electrochemical cycling, CV measurements were performed at a scan rate of 100 mV.s^{-1} under Ar-saturated H_2SO_4 electrolyte, systematically extending the negative potential limit. As shown in **Fig. S6a**, C- WO_3 exhibits three well-defined reductive peaks during the cathodic scan, beginning with the initial intercalation onset at ~0.32 V vs RHE (red arrow). Beyond -0.65 V, vigorous HER activity emerges, indicated by gas evolution.

The anodic scan reveals that increased negative potential leads to higher anodic currents, particularly for peak 1′, which also shifts positively in potential indicative of stronger W–H* binding and increased H^+ content within the lattice. This trend aligns with theoretical expectations of H-bond strengthening at deeper reduction levels.[6] For the 2% P-OV-WO_3 catalyst (**Fig. S6b**), hydrogen intercalation onset remains at 0.32 V vs RHE, but only the first peak is discernible, with peaks 2 and 3 absent. Furthermore, anodic peak 1' shows minimal shift with increasing cathodic bias, suggesting a fundamentally different intercalation/deintercalation mechanism and W–H* binding profile. Similar behaviors are observed for the 5% and 7.5% P-OV-WO_3 samples (**Fig. S6c and S6d**), though subtle differences in peak shape and intensity reflect nuanced effects of phosphorus concentration on hydrogen storage dynamics. These high-rate CV results reinforce the conclusion that phosphorus doping inhibits deep hydrogen intercalation and alters the redox behavior of W-H*, consistent with the trends observed under slow-scan conditions.

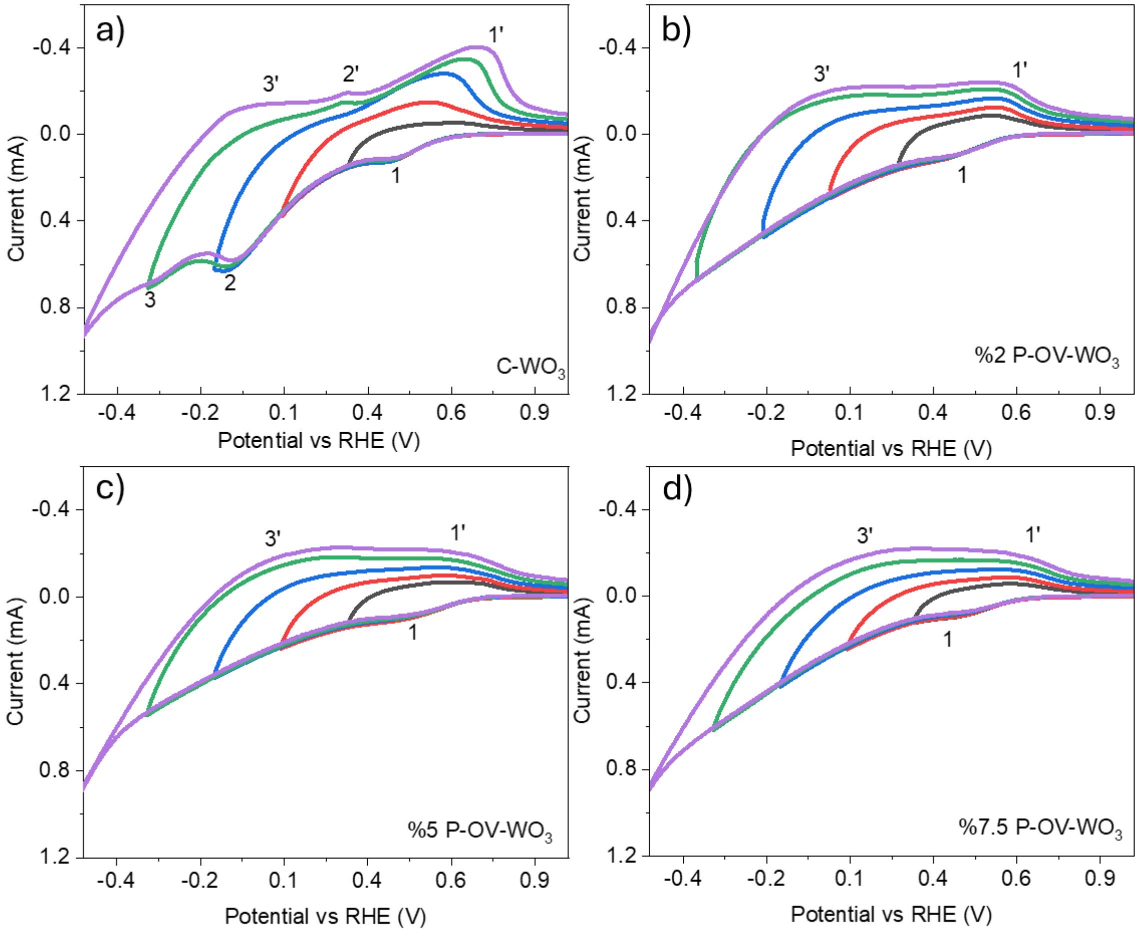

Figure S6. Cyclic Voltammograms (CV) of (a) C-WO_3, (b) %2 P-OV-WO_3, (c) %5 P-OV-WO_3, and (d) %7.5 P-OV-WO_3 with the cathodic potential gradually increase as interval of 0.200V, scan rate of 100 $mV.s^{-1}$.

S3.7 Stability test

To investigate the stability of P-OV-WO_3 electrocatalyst, a chronoamperometric durability measurement was performed on P-OV-WO_3 at -0.15 V vs RHE, a potential which is relevant to HER suppression analysis. Two independent measurements of tests were conducted using identically prepared electrodes to perform a relatively longer-term test (5 h) and a short-term control test (10 min). In both cases, the current remains below 0.1 mA, demonstrating stable HER suppression **(Figure S7a**). After each test, the electrodes were held at open-circuit potential (OCV)

for 1 h to recover the initial state of the catalysts, following by thoroughly rinsing and drying prior to X-ray diffraction (XRD) and X-ray photoelectron spectroscopy (XPS) characterization. All spectra were obtained after subtraction of the carbon paper background **(Figure S8)**.

For reference, the diffraction pattern of the as-prepared P-OV-WO_3 powder and the standard monoclinic WO_3 (PDF) were also included in **Figure S7b**. No discernible structural changes or phase transformations are observed after the chronoamperometric test. The major diffraction features remain aligned with those of the as-prepared P–OV–WO_3 powder, indicating that P-OV-WO_3 catalyst retains its crystallographic integrity under the applied conditions. These results confirm the robust structural stability of the catalyst within the HER-relevant potential window.

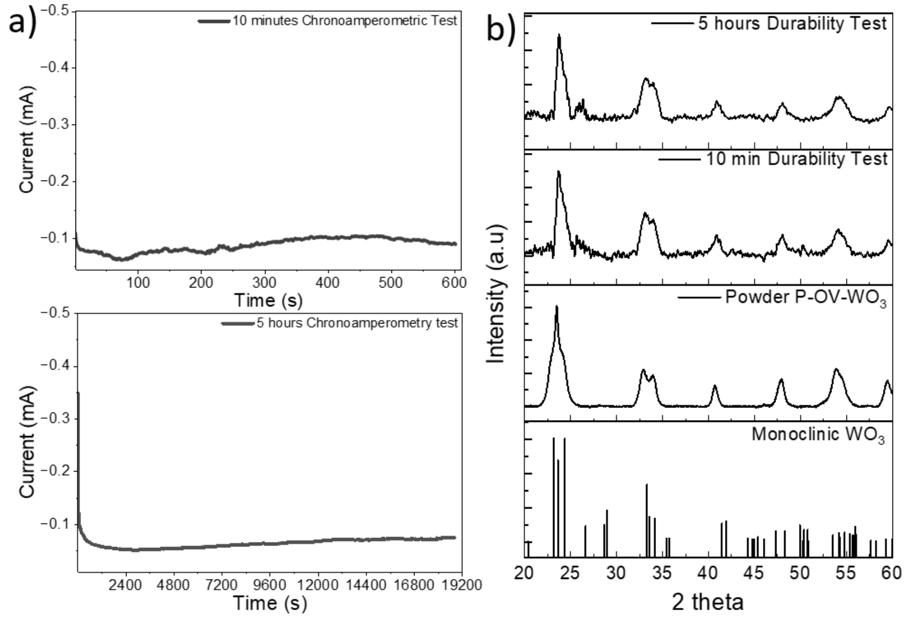

Figure S7. (a) Chronoamperometric I–t curves of P-OV-WO_3 catalyst deposited on carbon paper at -0.15 V vs RHE for 5 h and 10 min, respectively. (b) XRD patterns of P-OV-WO_3 catalyst after chronoamperometric polarization at -0.15 V vs RHE for 5 h and 10 min, respectively, with the carbon paper background subtracted. For comparison, the diffraction pattern of as-prepared powder P-OV-WO_3 and the reference PDF pattern of monoclinic WO_3 are also shown.

Following the electrochemical testing, XPS analysis was also conducted to evaluate possible changes in the chemical states of W, O, and P. As shown in **Figure S8**, the W 4f spectra retain the characteristic W^{6+}/W^{5+} components with no significant shift in binding energy or change in relative intensity, indicating preservation of the tungsten oxidation state distribution. The O 1s spectra shows consistent lattice oxygen (OL) and hydroxyl (OH^-) contributions, with no evidence of substantial restructuring or new oxygen species formation. The $P2p_{(3/2)}$ and $P2p_{(1/2)}$ doublets show no discernible change with the $P2p_{(3/2)}$ peak remaining at ~134 eV and the $P2p_{(1/2)}$ peak at ~135 eV in both datasets, indicating no reduction to P^0 or formation of new phosphorus phases. Together with the XRD results, these findings provide complementary evidence that both the surface chemistry and bulk crystal structure remain stable under HER-suppressing electrochemical conditions.

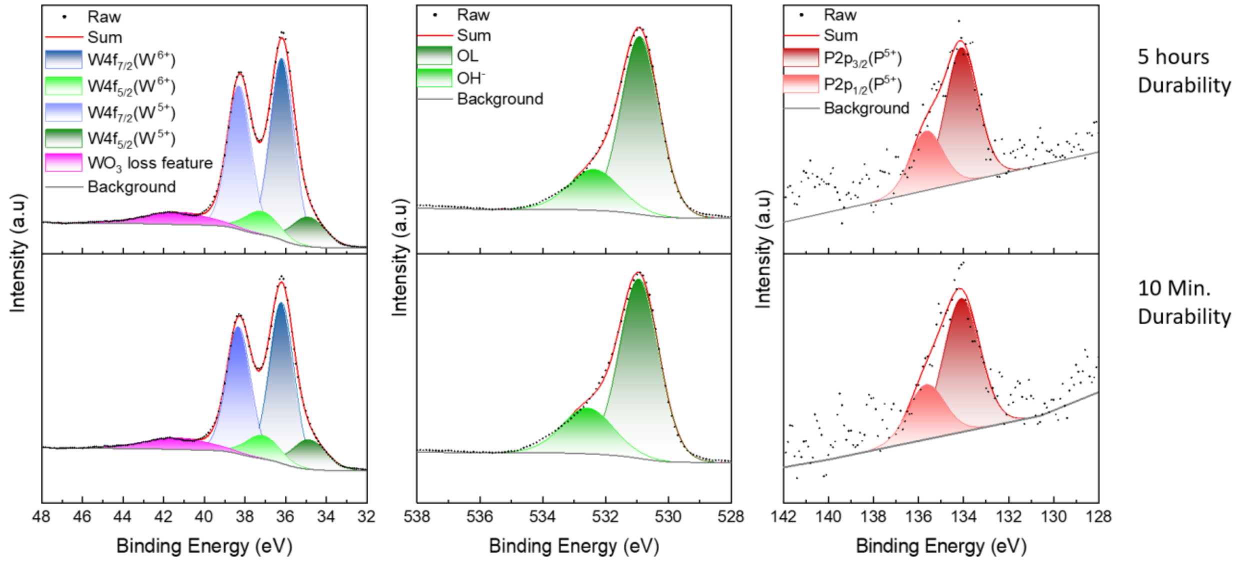

Figure S8. XPS spectra of P-OV-WO_3 after chronoamperometric testing at -0.15 V vs RHE for 10 min and 5 h: *(left)* W 4f, *(middle)* O 1s, and *(right)* P 2p regions. The consistent spectral features indicate preservation of W oxidation states, oxygen environment, and P(V) chemical state after both short- and long-term electrochemical operation.

S3.8 Electrochemical Impedance Spectroscopy (EIS) Studies: Nyquist Plots and Equivalent Circuit Fitting

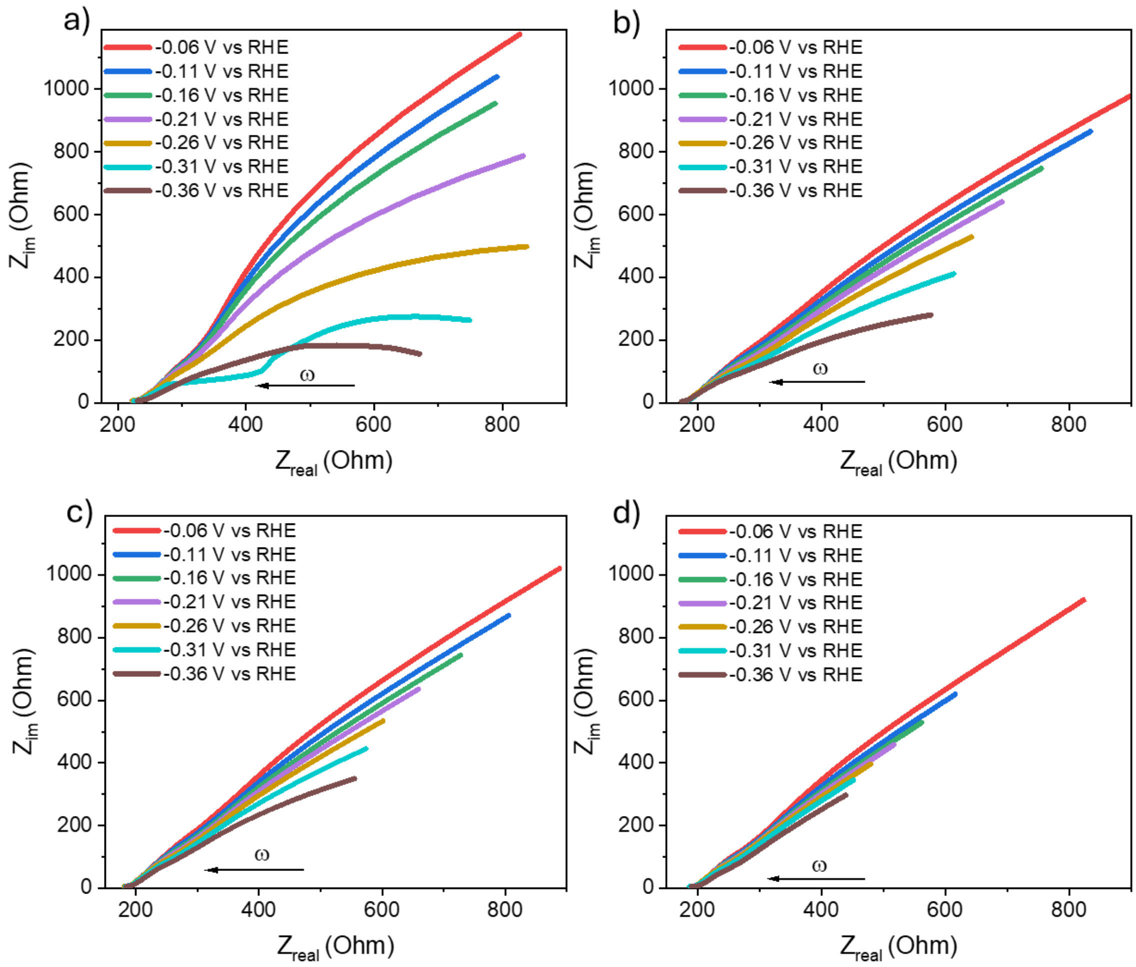

Figure S9. Nyquist plots of (a) C- WO_3, (b) %2 P-OV-WO_3, (c) %5 P-OV-WO_3, and (d) %7.5 P-OV-WO_3

Table S3-1. Nyquist-Plot Equivalent Circuit Fitting Parameters for C-WO_3 (Fig. S6a)

Potential vs RHE (V)	Rs (Ω)	Rct (Ω)	Ri (Ω)	Cq (F)	Rc (Ω)	Goodness of fit	Kramer Kronig
-0.06	215.50 (±) 3.30	95.53 (±) 2.60	1.413E+06 (±) 4.3E+05	2.127E-04 (±) 6.2E-06	116.10 (±) 0.59	5.99E-04 (±) 4.4E-07	4.28E-04
-0.11	216.40 (±) 3.60	95.94 (±) 2.50	1.737E+05 (±) 5.0E+03	2.170E-04 (±) 1.4E-05	114.60 (±) 1.40	5.775E-04 (±) 2.9E-07	4.18E-04
-0.16	226.40 (±) 3.80	93.01 (±) 2.70	4.243E+04 (±) 2.2E+04	2.403E-04 (±) 2.4E-06	111.8 (±) 0.69	5.19E-04 (±) 7.4E-06	3.98E-04
-0.21	224.10 (±) 2.30	94.91 (±) 4.10	6.575E+03 (±) 8.4E+02	2.607E-04 (±) 1.2E-05	109.2 (±) 2.30	4.775E-04 (±) 1.4E-05	3.81E-04

-0.26	223.40 (±) 3.30	95.94 (±) 2.00	1.860E+03 (±) 4.1E+01	3.087E-04 (±) 7.9E-06	106.7 (±) 0.21	4.261E-04 (±) 1.4E-05	3.33E-04

Table S3-2. Nyquist-Plot Equivalent Circuit Fitting Parameters for %2 P-OV-WO_3 (Fig. S6b)

Potential vs RHE (V)	Rs (Ω)	Rct (Ω)	Ri (Ω)	Cq (F)	Rc (Ω)	Goodness of fit	Kramer Kronig
-0.06	161.49 (±) 4.05	71.16 (±) 6.25	1.301E+06 (±) 1.4E+05	1.001E-04 (±) 1.1E-06	53.33 (±) 5.82	4.56E-04 (±) 1.5E-07	3.61E-04
-0.11	161.74 (±) 3.18	69.37 (±) 2.33	8.607E+05 (±) 2.2E+05	1.010E-04 (±) 3.0E-06	53.04 (±) 2.39	4.21E-04 (±) 6.3E-06	3.44E-04
-0.16	166.35 (±) 4.53	66.94 (±) 2.70	4.225E+05 (±) 6.4E+04	1.068E-04 (±) 1.2E-06	53.13 (±) 2.15	3.84E-04 (±) 1.2E-06	3.27E-04
-0.21	171.03 (±) 1.96	68.74 (±) 1.68	2.035E+05 (±) 1.3E+04	1.260E-04 (±) 5.0E-06	53.24 (±) 3.06	3.37E-04 (±) 8.7E-06	2.990E-04
-0.26	172.72 (±) 2.09	73.61 (±) 1.46	7.669E+04 (±) 5.0E+04	1.327E-04 (±) 6.4E-07	53.25 (±) 1.24	3.25E-04 (±) 2.2E-05	2.98E-04
-0.31	175.53 (±) 1.37	70.09 (±) 0.56	1.389E+04 (±) 4.4E+02	1.583E-04 (±) 3.1E-06	52.31 (±) 5.88	2.95E-04 (±) 5.4E-06	2.70E-04
-0.36	174.26 (±) 1.66	71.66 (±) 5.79	1.746E+03 (±) 1.0E+02	1.841E-04 (±) 2.8E-05	52.0 (±) 3.66	3.09E-04 (±) 5.6E-05	2.73E-04

Table S3-3. Nyquist-Plot Equivalent Circuit Fitting Parameters for %5 P-OV-WO_3 (Fig. S6c)

Potential vs RHE (V)	Rs (Ω)	Rct (Ω)	Ri (Ω)	Cq (F)	Rc (Ω)	Goodness of fit	Kramer Kronig
-0.06	169.93 (±) 1.14	68.94 (±) 2.78	3.725E+06 (±) 4.8E+05	1.101E-04 (±) 5.7E-06	57.31 (±) 1.68	4.64E-04 (±) 4.0E-05	3.98E-04
-0.11	169.36 (±) 8.01	65.59 (±) 1.18	1.024E+06 (±) 1.3E+05	1.143E-04 (±) 2.4E-06	58.04 (±) 1.19	4.25E-04 (±) 4.5E-07	4.29E-04
-0.16	160.04 (±) 1.72	64.57 (±) 1.35	8.231E+05 (±) 1.5E+05	1.181E-04 (±) 2.6E-06	58.16 (±) 1.55	3.83E-04 (±) 4.3E-05	3.44E-04
-0.21	169.90 (±) 1.22	64.83 (±) 1.69	4.956E+05 (±) 9.2E+04	1.233E-04 (±) 2.0E-06	58.76 (±) 5.27	3.43E-04 (±) 2.8E-06	3.16E-04
-0.26	169.77 (±) 2.64	62.30 (±) 2.81	2.166E+05 (±) 7.5E+04	1.432E-04 (±) 2.8E-06	57.85 (±) 6.14	3.06E-04 (±) 7.1E-06	2.88E-04
-0.31	170.72 (±) 1.12	70.08 (±) 2.32	2.748E+04 (±) 3.1E+03	1.583E-04 (±) 9.2E-06	56.58 (±) 1.24	2.42E-04 (±) 2.4E-05	2.222E-04
-0.36	170.68 (±) 1.14	71.73 (±) 3.70	4.732E+03 (±) 4.7E+01	1.731E-04 (±) 5.8E-08	55.37 (±) 1.48	2.316E-04 (±) 4.6E-05	2.28E-04

Table S3-4. Nyquist-Plot Equivalent Circuit Fitting Parameters for %7.5 P-OV-WO_3 (Fig. S6d)

Potential vs RHE (V)	Rs (Ω)	Rct (Ω)	Ri (Ω)	Cq (F)	Rc (Ω)	Goodness of fit	Kramer Kronig
-0.06	172.68 (±) 2.17	65.59 (±) 9.06	3.291E+06 (±) 1.3E+06	9.394E-05 (±) 1.1E-05	57.87 (±) 6.44	4.271E-04 (±) 6.6E-06	4.09E-04
-0.11	171.52 (±) 2.83	62.23 (±) 3.94	1.605E+06 (±) 5.8E+05	1.000E-04 (±) 0.0E+00	57.69 (±) 1.46	3.795E-04 (±) 8.8E-06	3.652E-04
-0.16	168.54 (±) 3.24	63.70 (±) 0.98	1.472E+06 (±) 2.7E+05	1.267E-04 (±) 1.6E-06	58.18 (±) 2.02	3.33E-04 (±) 5.8E-04	3.20E-04
-0.21	172.88 (±) 3.04	63.91 (±) 3.08	7.803E+05 (±) 9.4E+04	1.355E-04 (±) 1.6E-06	57.66 (±) 1.23	2.92E-04 (±) 9.8E-04	2.820E-04
-0.26	176.11 (±) 2.56	64.79 (±) 8.04	2.507E+05 (±) 1.9E+04	1.417E-04 (±) 2.9E-06	58.14 (±) 0.02	2.60E-04 (±) 0.0E+00	2.480E-04
-0.31	168.77 (±) 2.91	66.61 (±) 1.44	5.760E+04 (±) 3.6E+04	1.699E-04 (±) 9.0E-06	57.57 (±) 2.54	2.275E-04 (±) 7.5E-04	2.155E-04
-0.36	172.76 (±) 3.49	66.52 (±) 1.79	8.272E+04 (±) 2.9E+04	1.715E-04 (±) 8.7E-06	57.60 (±) 5.82	1.99E-04 (±) 6.6E-06	1.80E-04

Capacitance Fitting of Constant Phase Elements

The equivalent electrical circuit used to fit the impedance spectra (Fig. 5) consists of two constant phase elements (CPE) representing distinct electrochemical regions: CPE1, assigned to the *electrolyte–catalyst interface*, and CPE2, corresponding to the *catalyst inner layer*. The true capacitances of these regions, denoted as Capacitance 1 (C1) for CPE1 and Capacitance 2 (C2) for CPE2 were calculated according to Eq.1[7, 8]

$$C = Yo^{1/\alpha}\left(R^{\frac{1-\alpha}{\alpha}}\right) \qquad \text{(Eq.1)}$$

where Yo with a unit of $\Omega^{-1}s^{\alpha}$, represents the pre-factor of the constant phase element (CPE), quantifying the strength of the non-ideal interfacial capacitive response arising from surface heterogeneity, defect-induced disorder, and distributed proton adsorption/intercalation kinetics. The parameter α quantifies the deviation of the constant phase element (CPE) from ideal capacitive behavior and is determined by fitting; α ranges from 0 to 1, with α = 1 representing an ideal

capacitor. R(Ω) is the resistance in parallel with the corresponding CPE element. C is the corrected or "true" capacitance (F) obtained from the non-ideal CPE response.

For CPE1, parameters Yo1 and α1 were extracted from fitting, and (1/Rct)+(1/Ri) (Ω) was used as the corresponding resistance. For CPE2, parameters Yo2 and α2 were extracted from fitting, with Rc (Ω) representing the inner-layer resistance. All parameters were obtained by nonlinear least-squares fitting using Gamry Echem Analyst software.

Table S4-1. Capacitance Fitting for C-WO_3 Using Equation [1]

Potential RHE (V)	(1/Rct)+(1/Ri) (W)	Yo1 ($\Omega^{-1}s^{\alpha}$)	a1	Capacitance 1 (F)	Rc (Ω)	Yo2 ($\Omega^{-1}s^{\alpha}$)	a2	Capacitance 2 (F)
**-0.06**	1.14E-02	7.41E-04	6.46E-01	1.23E-06	1.16E+02	1.03E-06	5.38E-01	4.33E-10
**-0.11**	1.04E-02	8.59E-04	6.44E-01	1.39E-06	1.15E+02	9.96E-07	5.38E-01	4.16E-10
**-0.16**	1.08E-02	8.95E-04	6.29E-01	9.86E-07	1.12E+02	6.87E-07	5.65E-01	4.63E-10
**-0.21**	1.07E-02	9.10E-04	5.98E-01	3.89E-07	1.09E+02	3.03E-07	6.38E-01	8.67E-10
**-0.26**	1.10E-02	8.94E-04	5.76E-01	1.84E-07	1.07E+02	1.17E-07	6.94E-01	8.13E-10

Table S4-2. Capacitance Fitting for %2 P-OV-WO_3 Using Equation [1]

Potential RHE (V)	(1/Rct)+(1/Ri) (Ω)	Yo1 ($\Omega^{-1}s^{\alpha}$)	α1	Capacitance 1 (F)	Rc (Ω)	Yo2 ($\Omega^{-1}s^{\alpha}$)	α2	Capacitance 2 (F)
**-0.06**	7.12E+01	1.30E+06	1.41E-02	1.21E-07	5.33E+01	1.21E-07	1.73E+02	3.00E-09
**-0.11**	6.94E+01	8.61E+05	1.44E-02	1.15E-07	5.33E+01	1.15E-07	1.73E+02	3.00E-09
**-0.16**	6.69E+01	4.23E+05	1.49E-02	1.08E-07	5.33E+01	1.08E-07	1.73E+02	3.00E-09
**-0.21**	6.87E+01	2.04E+05	1.46E-02	7.33E-08	5.32E+01	7.33E-08	1.72E+02	3.08E-09
**-0.26**	7.36E+01	7.67E+04	1.36E-02	4.16E-08	5.33E+01	4.16E-08	1.72E+02	3.02E-09
**-0.31**	7.01E+01	1.39E+04	1.43E-02	8.67E-09	5.27E+01	8.67E-09	1.72E+02	2.68E-09
**-0.36**	7.17E+01	1.75E+03	1.45E-02	4.55E-11	5.20E+01	1.93E-08	1.70E+02	2.56E-09

Table S4-3. Capacitance Fitting for %5 P-OV-WO_3 Using Equation [1]

Potential RHE (V)	(1/Rct)+(1/Ri) (Ω)	Yo1 ($\Omega^{-1}s^{\alpha}$)	α1	Capacitance 1 (F)	Rc (Ω)	Yo2 ($\Omega^{-1}s^{\alpha}$)	α2	Capacitance 2 (F)
**-0.06**	1.45E-02	8.53E-04	5.69E-01	1.64E-07	5.74E+01	5.88E-08	7.85E-01	1.87E-09
**-0.11**	1.52E-02	9.99E-04	5.63E-01	1.82E-07	5.80E+01	1.42E-07	6.85E-01	6.55E-10
**-0.16**	1.55E-02	1.17E-03	5.56E-01	1.91E-07	5.82E+01	1.54E-07	6.81E-01	6.57E-10
**-0.21**	1.54E-02	1.37E-03	5.52E-01	2.20E-07	5.88E+01	1.96E-07	6.64E-01	6.16E-10
**-0.26**	1.61E-02	1.60E-03	5.42E-01	1.59E-07	5.79E+01	2.03E-07	6.62E-01	6.21E-10
**-0.31**	1.43E-02	1.82E-03	5.18E-01	9.87E-08	5.67E+01	7.60E-08	7.41E-01	1.02E-09
**-0.36**	1.42E-02	2.00E-03	5.03E-01	6.44E-08	5.55E+01	1.11E-07	6.99E-01	6.31E-10

Table S4-4. Capacitance Fitting for %7.5 P-OV-WO_3 Using Equation [1]

Potential RHE (V)	(1/Rct)+(1/Ri) (Ω)	Yo1 ($\Omega^{-1}s^{\alpha}$)	α1	Capacitance 1 (F)	Rc (Ω)	Yo2 ($\Omega^{-1}s^{\alpha}$)	α2	Capacitance 2 (F)
**-0.06**	1.52E-02	7.39E-04	5.65E-01	1.14E-07	5.79E+01	8.15E-09	9.21E-01	2.35E-09
**-0.11**	1.61E-02	7.28E-04	5.54E-01	7.81E-08	5.78E+01	7.42E-09	9.28E-01	2.38E-09
**-0.16**	1.57E-02	9.13E-04	5.38E-01	6.33E-08	5.82E+01	8.36E-09	9.17E-01	2.26E-09
**-0.21**	1.56E-02	1.01E-03	5.36E-01	7.03E-08	5.78E+01	8.27E-09	8.26E-01	3.89E-10
**-0.26**	1.54E-02	1.13E-03	5.34E-01	7.94E-08	5.81E+01	8.57E-09	9.15E-01	2.23E-09
**-0.31**	1.50E-02	1.23E-03	5.18E-01	4.84E-08	5.76E+01	6.56E-09	9.37E-01	2.42E-09
**-0.36**	1.50E-02	1.37E-03	5.04E-01	3.34E-08	5.76E+01	7.16E-09	9.24E-01	2.13E-09

S3.9 Hydrogen bond dissociation free energy (H-BDFE) measurements

Following the reported methodology,[9] the H-BDFE on C-WO_3 and P-OV-WO_3 catalysts were quantified. Briefly, cyclic voltammetry (CV) measurements were conducted on C-WO_3 and P-OV-WO_3 catalysts deposited on the glassy carbon disc electrodes in Ar-saturated aqueous electrolytes (pH 1-3). Catalyst ink preparation and electrode deposition followed the same procedures described in **Section S2.1**. CVs were recorded at a scan rate of 5 mV s^{-1} using an

Ag/AgCl reference electrode, and all potentials were converted to the standard hydrogen electrode (SHE) scale according to: $E_{SHE} = E_{Ag/AgCl} + 0.197$ V

For both catalysts, the first intercalation and the corresponding deintercalation peaks shifted negatively, consistent to previous reports.[10] The half-wave potentials ($E_{1/2}$) were determined from the average of anodic and cathodic peak potentials extracted from the CV profiles (**Figure S8a, c).** The pH dependence of $E_{1/2}$ for the first peak is shown in **Figure S8b, d.** Notably, the second intercalation peak for P-OV-WO_3 catalyst is largely diminished due to the largely suppressed proton intercalation. Therefore, the H–BDFE was estimated based on the first intercalation/deintercalation peak and used to compare the relative thermodynamic properties of the two catalysts.

Linear fits yield slopes of 59.8 ±0.96 mV/pH for C-WO_3 and 59.0 ±0.96 mV/pH for 5% P-OV-WO_3. These slopes closely match the theoretical Nernstian value (59 mV/pH), consistent with a $1H^+/1e^-$ PCET mechanism for the intercalation/deintercalation reactions. The standard half-wave potentials ($E^0_{1/2}$, obtained by extrapolation to pH = 0) were used to estimate the H bond dissociation free energy (BDFE) using: H-BDFE = $52.8+23.06\times E^0_{1/2}$

The H-BDFE values are 54.9±1.22 kcal/mol for P-OV-WO_3 and 57.2±0.78 kcal/mol for C-WO_3**.** The lower H-BDFE of P-OV-WO_3 indicates a thermodynamically weaker H atom interaction with the catalyst, which facilitates hydrogen atom transfer to reactant molecules, and thus favors hydrogenation reactions. Importantly, HER activity is not governed solely by H-BDFE but is strongly dependent on H* surface coverage. In our system, P-OV-WO_3 exhibits reduced H* coverage, which kinetically suppress H_2 formation despite the lower BDFE.

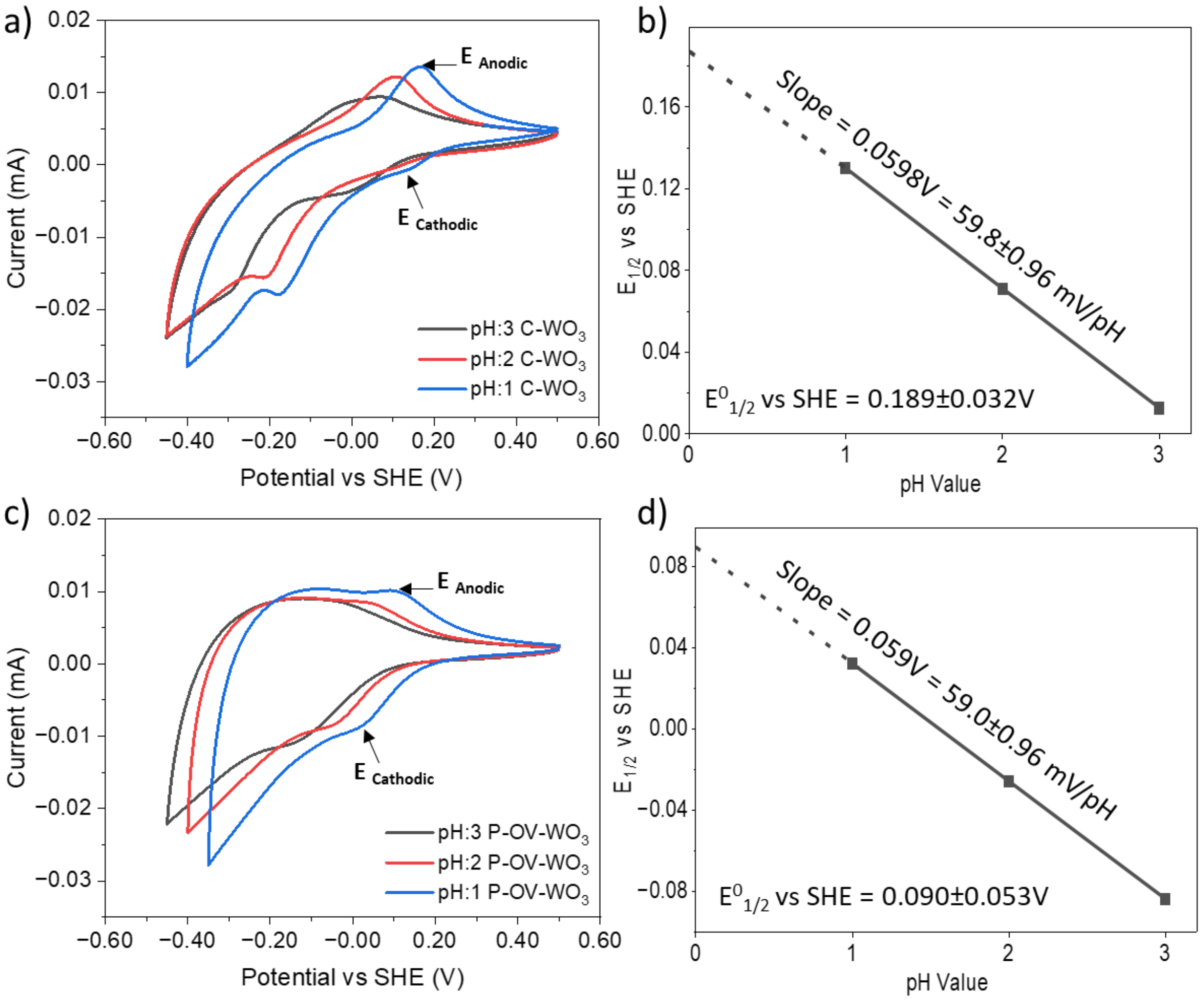

Figure S10. Cyclic voltammograms of C-WO_3 *(a)* and P-OV-WO_3 *(c)* recorded in electrolytes with different pH values (pH 1–3). *(b, d)* Linear dependence of the first midpoint potentials ($E_{1/2}$) vs pH for C-WO_3 *(b)* and P-OV-WO_3 *(d)*, exhibiting slopes of ~58 mV/pH, consistent with a proton-coupled electron transfer process. The extracted $E°_{1/2}$ vs SHE was used to estimate the H bond dissociation free energies.

S4 Computational Details

Following the work of Miu et al.,[11] we used the Vienna Ab-initio Simulation Package (VASP) [12] to perform periodic Kohn−Sham DFT calculations utilizing the Perdew−Burke−Ernzerhof (PBE) generalized gradient approximation (GGA) for the XC functional.[13] We used the VASP-supplied projector augmented-wave potentials for the (frozen) core−valence electron interactions.[13] We

imposed a kinetic-energy cutoff of 520 eV for the planewave basis set. All calculations were performed using a 3´3´3 k-point mesh to sample the reciprocal space within the Monkhorst−Pack method.[14] Electronic energies were converged to variations of less than 1×10^{-6} eV and relaxations were conducted using the conjugate gradient algorithm until all forces became smaller than 0.01 eV/Å. The dispersion corrections were accounted for using Grimme's D3 correction[15] with Becke−Johnson damping. Dispersion correction combined with PBE functional has been shown to improve predictions for formation energies[16] and metal oxide lattice parameters[15] as compared to PBE calculations without dispersion correction. Additionally, we also found that there was no discernible difference between spin-polarized and non-spin-polarized calculations for representative calculations of the system under consideration. Please note that PBE and other GGA class of functionals are known to underestimate bandgaps.[16] One of the solutions to these limitations is to use hybrid functionals such as HSE06, which demonstrate better bandgap prediction. However, the calculation cost of using hybrid functionals such as HSE06 is much higher than that of using PBE functionals. Additionally, Miu et al.[11] demonstrated that using HSE06 instead of PBE primarily results in a constant offset in energies for intercalated or non-intercalated WO_3 systems. Hence, PBE functional-based calculations should be able to capture the trends in electronic structure changes due to intercalation. In order to balance computational cost and accuracy, we employ PBE-D3 calculations in this work.

All the calculations were performed starting with a monoclinic unit cell of WO_3 containing eight W-atoms and 24 O-atoms corresponding to Materials project structure ID of mp-619461. The following **Table S5** shows a comparison between experimental[17] and DFT optimized lattice parameters after geometry optimization of the unit cell. The following **Table S5** shows a comparison between experimental[17] and DFT optimized lattice parameters after geometry

optimization of the unit cell. The optimized WO_3 unit cell, hereafter referred to as C-WO_3 was then used to create P-OV-WO_3 system, where an O-vacancy was created by removing an O-atom from c-WO_3 cell and then replacing W-atom next to the vacancy by a P-atom. The P-OV-WO_3 cell was also optimized before performing H-intercalation calculations. **Fig. S12** shows the optimized structures for both C-WO_3 and P-OV-WO_3 respectively. For modelling first H-intercalation, an H-atom was placed next to every O-atom in the respective until cells and then the structure was optimized while keeping the cell dimensions constant. This resulted in 24 and 23 H-intercalation calculations for C-WO_3 and P-OV-WO_3 respectively. Proton intercalation in oxide materials can be computationally studied through several approaches, including static adsorption energy calculations for thermodynamic stability and nudged elastic band calculations for migration barriers. In this work, we focus on adsorption energies of protons at bulk interstitial sites, as these directly determine the thermodynamic driving force for intercalation and capture how P-doping modifies the energetic landscape for proton accommodation. Kinetic barrier calculations to assess how P-doping affects proton migration rates represent an important direction for future work

Table S5: Comparison of experimental and DFT relaxed bulk WO_3 lattice parameters.

**Method**	**a (Å)**	**b (Å)**	**c (Å)**	**α**	**β**	**γ**
This work	7.71	7.68	7.72	90°	90.19°	90°
Experimental [17]	7.30	7.53	7.68	90°	90.54°	90°

The H-intercalated structures were then analyzed to calculate hydrogen binding energy, density of states (DOS) as well as partial charges as a function of first H-intercalation. The H-binding energy is defined as:

$$\Delta\ \mathrm{E}_{\mathrm{H-bind}} = \mathrm{E}_{\mathrm{H_{0.125}WO_3(w/o\ P-doping)}} - \mathrm{E}_{\mathrm{WO_3(w/o\ P-doping)}} - 0.5\mathrm{E}_{\mathrm{H_2}}$$

Local and projected electronic density of states (DOS) were generated for each optimized structure with the occupied and unoccupied electronic band centers determined from the relevant local, projected DOS. The partial charges were determined using Bader charge analysis using the Henkelman method.[18] The calculated H-binding energies were also used to calculate equilibrium electrochemical potentials for first H-intercalation by applying the computational hydrogen electrode (CHE) model.[19] The potential was calculated using the following equation similar to the work of Miu et al.[11]:$\Delta E_{int} = \Delta E_{H,WO_3,w/o\ P-doping,\ expected} - E_{WO_3,\ w/o\ P-doping,\ expected} - eU$Local and projected electronic density of states (DOS) were generated for each optimized structure with the occupied and unoccupied electronic band centers determined from the relevant local, projected DOS. The partial charges were determined using Bader charge analysis using the Henkelman method.[18] The calculated H-binding energies were also used to calculate equilibrium electrochemical potentials for first H-intercalation by applying the computational hydrogen electrode (CHE) model.[19] The potential was calculated using the following equation similar to the work of Miu et al.[19]

$$\Delta E_{int} = \Delta E_{H,WO_3,w/o\ P-doping,\ expected} - E_{WO_3,\ w/o\ P-doping,\ expected} - eU$$

where the charge of the electron is denoted by e and the applied electric potential by U. The value of U when $\Delta E_{int} = 0$ is taken as the equilibrium electrochemical hydrogen intercalation potential. This potential can be directly compared to the empirically observed equilibrium potential versus the reversible hydrogen electrode (RHE).[11]where the charge of the electron is denoted by e and the applied electric potential by U. . The value of U when $\Delta E_{int} = 0$ is taken as the equilibrium electrochemical hydrogen intercalation potential. This potential can be directly compared to the empirically observed equilibrium potential versus the reversible hydrogen electrode (RHE).[11]

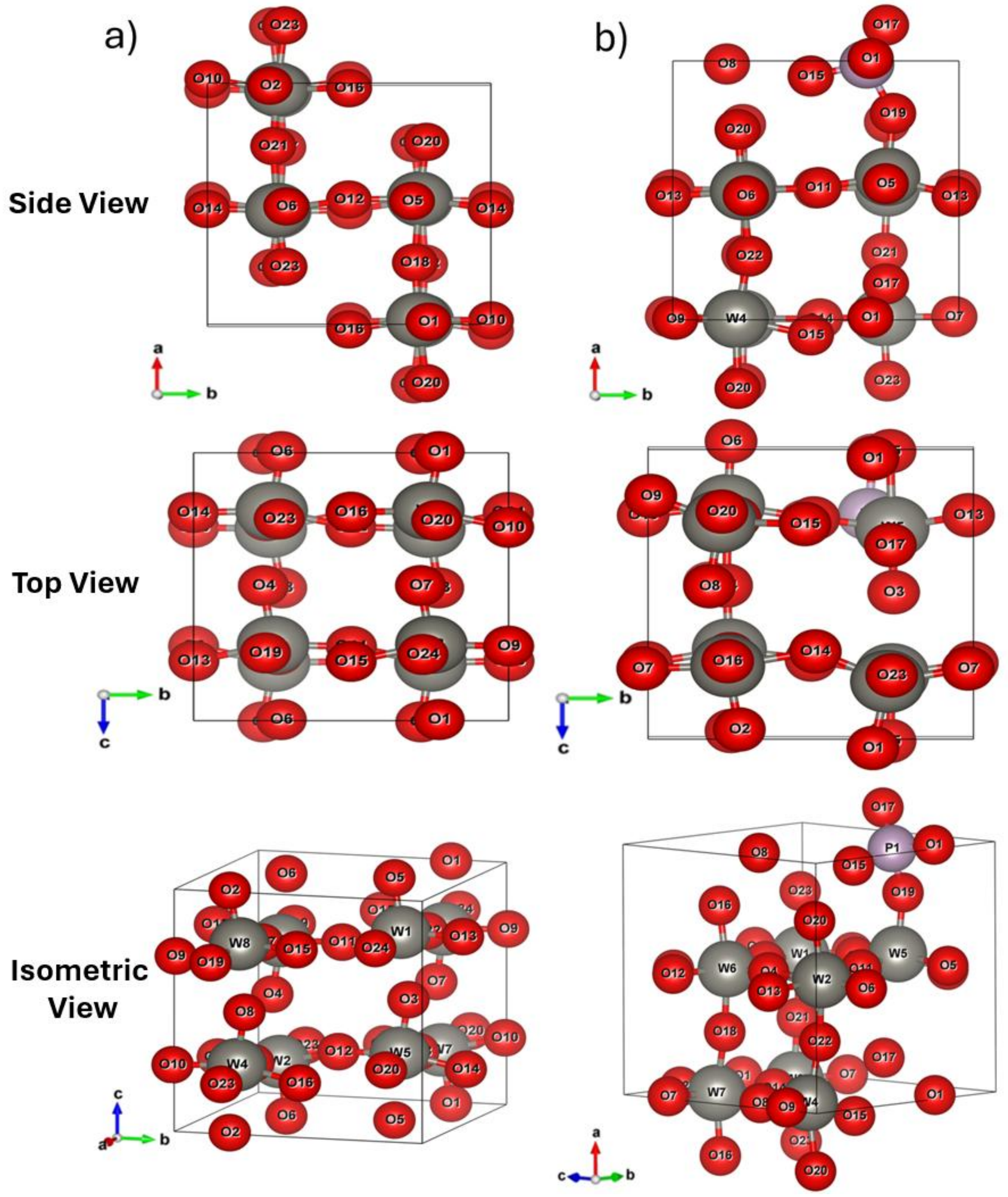

Figure S11. DFT optimized structures of a) C-WO_3 and b) P-OV-WO_3 models. Red spheres represent O-atoms, grey spheres show W-atoms, and pink sphere shows P-atom. All the atom sites are numbered and superimposed on respective atom spheres.

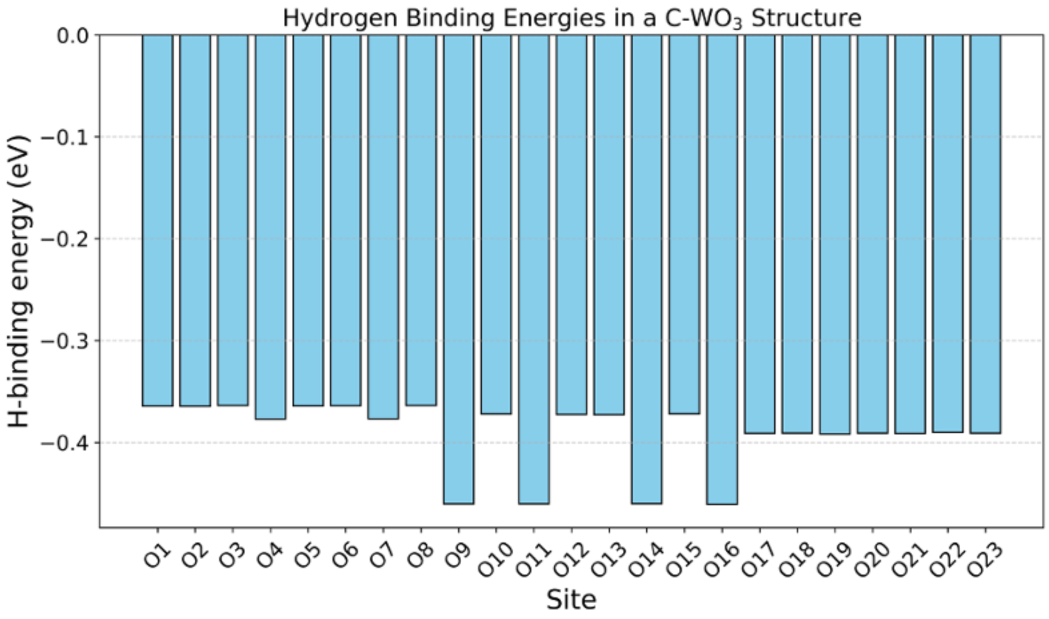

Figure S12. H-binding energy in eV on all O-sites in a C-WO_3 structure. The site labels refer to the O-atom sites illustrated in Fig. **S11A**.

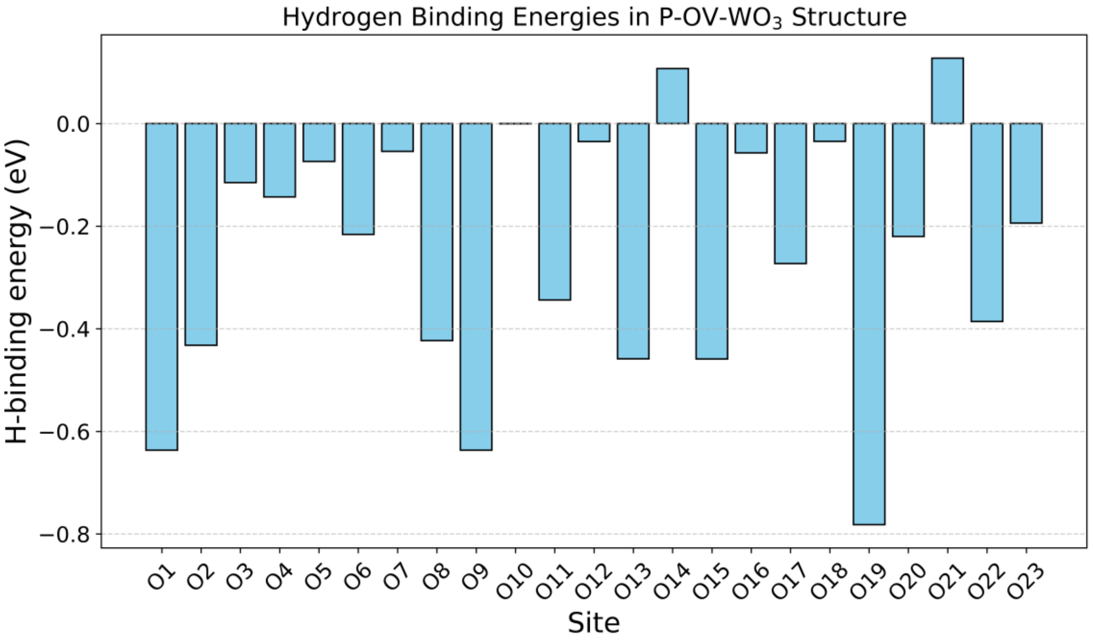

Figure S13. H-binding energy in eV on all O-sites in a P-OV-WO_3 structure. The site labels refer to the O-atom sites illustrated in Fig. **S11B**.

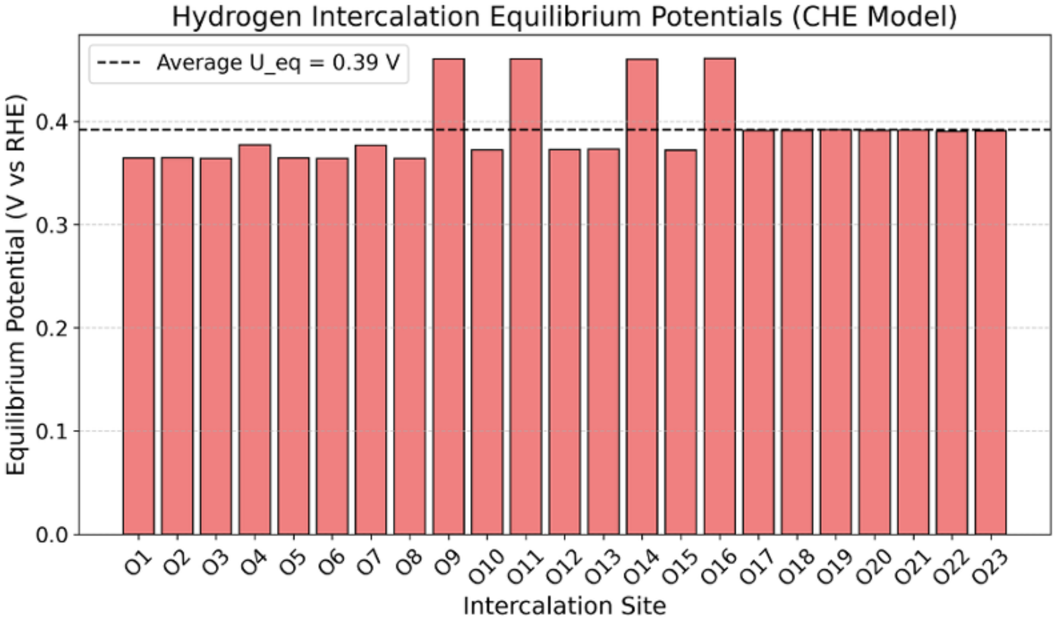

Figure S14. H-intercalation equilibrium potentials for all O-sites in a C-WO_3 structure. The site labels refer to the O-atom sites illustrated in **Fig. S11**A.

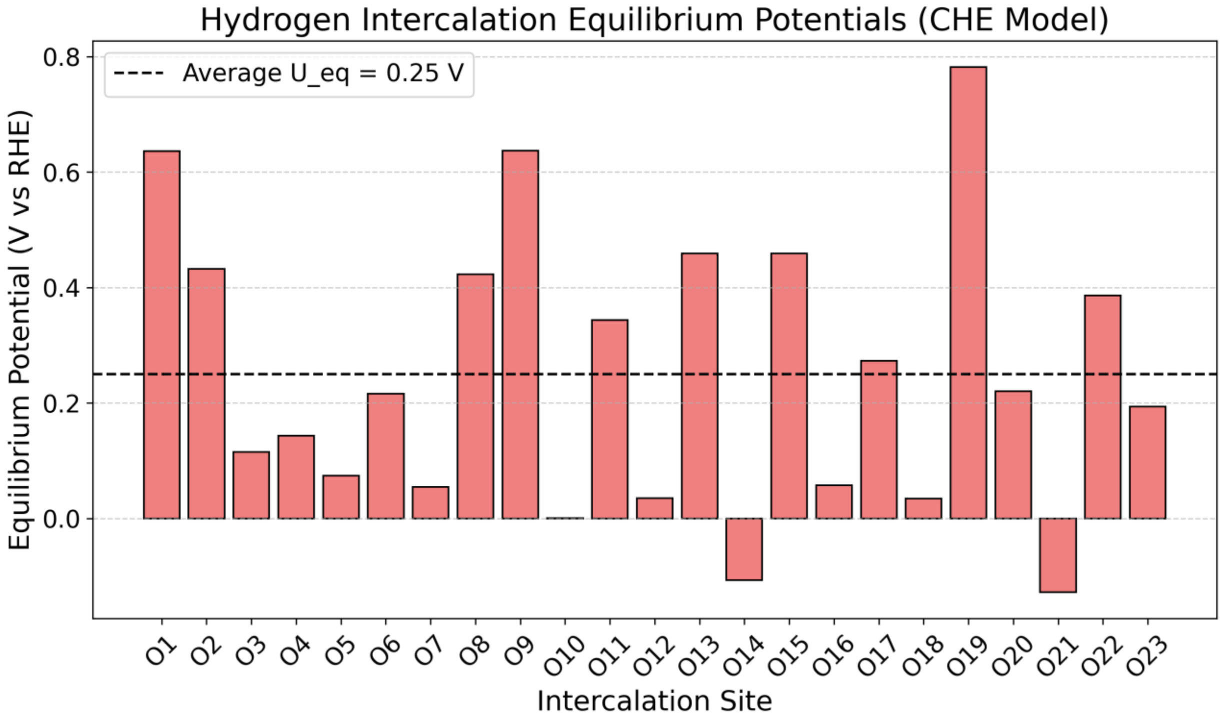

Figure S15. H-intercalation equilibrium potentials for all O-sites in a P-OV-WO_3 structure. The site labels refer to the O-atom sites illustrated in **Fig. S11B**.

S5. Benchmark discussion of PBE-D3 vs HSE06

To assess the validity of our PBE-based conclusions, we performed benchmark calculations using the HSE06 hybrid functional on representative C-WO_3 and P-doped WO_3 structures. The experimental band gap for cubic WO_3 is 2.69 eV, while our PBE-D3 calculations yield 1.37 eV and HSE06 calculations yield 2.32 eV. This confirms the known underestimation of band gaps by PBE, with HSE06 providing closer agreement with experiment. As shown in **Figure S16**, the primary difference between PBE and HSE06 results is a rigid shift in band positions, consistent with the known underestimation of band gaps by PBE. Crucially, the qualitative trends (or shapes) in the density of states upon P-doping are preserved in both functionals. This confirms that our mechanistic conclusions regarding electronically mediated change in proton intercalation upon doping are robust with respect to functional choice.

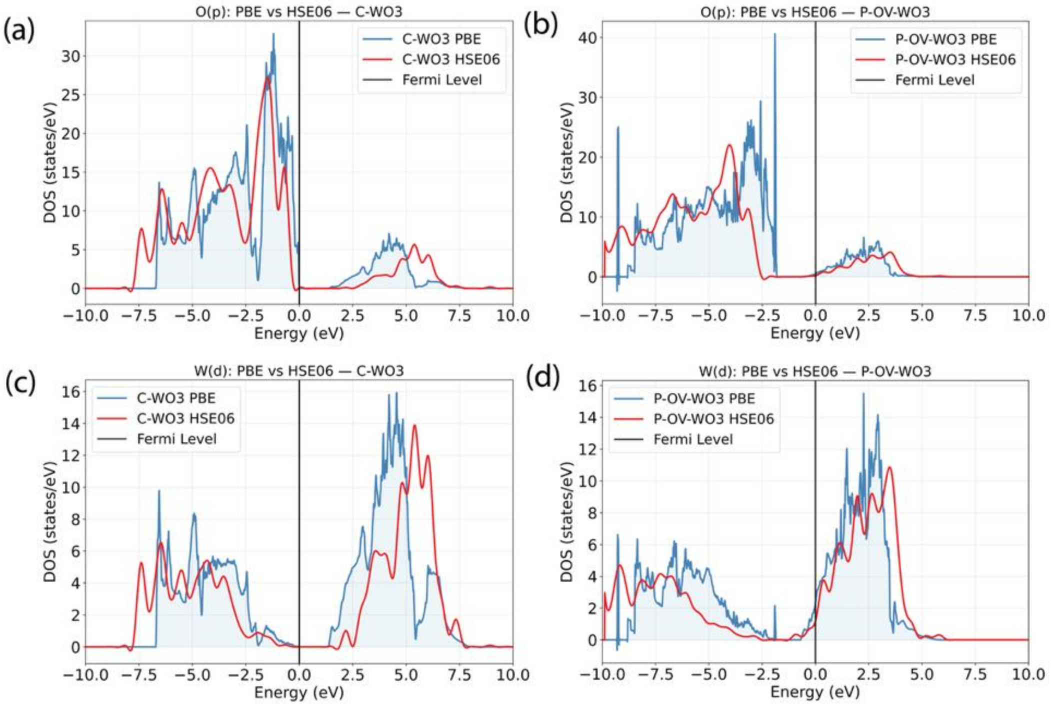

Figure S16. Comparison of electronic density of states with O p-band and W d-band for C-WO_3 and P-OV-WO_3 using PBE-D3 and HSE06 methods.

REFERENCES

(1) Chen, R.; Yang, C.; Cai, W.; Wang, H.-Y.; Miao, J.; Zhang, L.; Chen, S.; Liu, B. Use of Platinum as the Counter Electrode to Study the Activity of Nonprecious Metal Catalysts for the Hydrogen Evolution Reaction. *ACS Energy Letters* **2017**, *2* (5), 1070–1075. DOI: 10.1021/acsenergylett.7b00219.
(2) Hasan, M. M.; Allam, N. K. An alternative, low-dissolution counter electrode to prevent deceptive enhancement of HER overpotential. *Scientific Reports* **2022**, *12* (1), 9368. DOI: 10.1038/s41598-022-13385-w.
(3) Li, Q.; Kucukosman, O. K.; Ma, Q.; Ouyang, J.; Kucheryavy, P.; Gu, H.; Long, C. L.; Zhang, Z.; Young, J.; Lockard, J. V.; et al. Enhancement of Electrochemical Nitrogen Reduction Activity and Suppression of Hydrogen Evolution Reaction for Transition Metal Oxide Catalysts: The Role of Proton Intercalation and Heteroatom Doping. *ACS Catalysis* **2024**, 8899–8912. DOI: 10.1021/acscatal.4c00223.
(4) Ke, J.; Zhou, H.; Liu, J.; Duan, X.; Zhang, H.; Liu, S.; Wang, S. Crystal transformation of 2D tungstic acid H2WO4 to WO3 for enhanced photocatalytic water oxidation. *Journal of Colloid and Interface Science* **2018**, *514*, 576–583. DOI: https://doi.org/10.1016/j.jcis.2017.12.066.
(5) Terohid, S. A. A.; Heidari, S.; Jafari, A.; Asgary, S. Effect of growth time on structural, morphological and electrical properties of tungsten oxide nanowire. *Applied Physics A* **2018**, *124* (8), 567. DOI: 10.1007/s00339-018-1955-0.
(6) Miu, E. V.; McKone, J. R.; Mpourmpakis, G. The Sensitivity of Metal Oxide Electrocatalysis to Bulk Hydrogen Intercalation: Hydrogen Evolution on Tungsten Oxide. *Journal of the American Chemical Society* **2022**, *144* (14), 6420–6433. DOI: 10.1021/jacs.2c00825.
(7) Gateman, S. M.; Gharbi, O.; Gomes de Melo, H.; Ngo, K.; Turmine, M.; Vivier, V. On the use of a constant phase element (CPE) in electrochemistry. *Current Opinion in Electrochemistry* **2022**, *36*, 101133. DOI: https://doi.org/10.1016/j.coelec.2022.101133.
(8) Negahdar, L.; Zeng, F.; Palkovits, S.; Broicher, C.; Palkovits, R. Mechanistic Aspects of the Electrocatalytic Oxygen Evolution Reaction over Ni−Co Oxides. *ChemElectroChem* **2019**, *6* (22), 5588–5595. DOI: https://doi.org/10.1002/celc.201901265.
(9) Ingram, Z. J.; Lander, C. W.; Oliver, M. C.; Altınçekiç, N. G.; Huang, L.; Shao, Y.; Noh, H. Hydrogen Atom Binding Energy of Structurally Well-Defined Cerium Oxide Nodes at the Metal–Organic Framework–Liquid Interfaces. *The Journal of Physical Chemistry C* **2024**, *128* (23), 9556–9565. DOI: 10.1021/acs.jpcc.4c02409.
(10) Holzapfel, N. P.; Papamatthaiakis, N. E.; Paudel, J. R.; Mpourmpakis, G.; Crumlin, E. J.; Augustyn, V. Effects of heteroatom doping on hydrogen uptake in tungsten oxide. *Chemical Science* **2026**, 10.1039/D5SC08564K. DOI: 10.1039/D5SC08564K.
(11) Miu, E. V.; Mpourmpakis, G.; McKone, J. R. Predicting the Energetics of Hydrogen Intercalation in Metal Oxides Using Acid–Base Properties. *ACS Applied Materials & Interfaces* **2020**, *12* (40), 44658–44670. DOI: 10.1021/acsami.0c11300.
(12) Kresse, G.; Furthmüller, J. Efficient iterative schemes for ab initio total-energy calculations using a plane-wave basis set. *Physical Review B* **1996**, *54* (16), 11169–11186. DOI: 10.1103/PhysRevB.54.11169. Kresse, G.; Joubert, D. From ultrasoft pseudopotentials to the projector augmented-wave method. *Physical Review B* **1999**, *59* (3), 1758–1775. DOI: 10.1103/PhysRevB.59.1758.
(13) Perdew, J. P.; Burke, K.; Ernzerhof, M. Generalized Gradient Approximation Made Simple. *Physical Review Letters* **1996**, *77* (18), 3865–3868. DOI: 10.1103/PhysRevLett.77.3865.
(14) Monkhorst, H. J.; Pack, J. D. Special points for Brillouin-zone integrations. *Physical Review B* **1976**, *13* (12), 5188–5192. DOI: 10.1103/PhysRevB.13.5188.

(15) Grimme, S.; Antony, J.; Ehrlich, S.; Krieg, H. A consistent and accurate ab initio parametrization of density functional dispersion correction (DFT-D) for the 94 elements H-Pu. *The Journal of Chemical Physics* **2010**, *132* (15), 154104. DOI: 10.1063/1.3382344 (acccessed 1/13/2026).
(16) Lee, Y.; Lee, T.; Jang, W.; Soon, A. Unraveling the Intercalation Chemistry of Hexagonal Tungsten Bronze and Its Optical Responses. *Chemistry of Materials* **2016**, *28* (13), 4528–4535. DOI: 10.1021/acs.chemmater.5b03980.
(17) Tanisaki, S. On the Phase Transition of Tungsten Trioxide below Room Temperature. *Journal of the Physical Society of Japan* **1960**, *15* (4), 566–573. DOI: 10.1143/JPSJ.15.566 (acccessed 2025/11/24).
(18) Tang, W.; Sanville, E.; Henkelman, G. A grid-based Bader analysis algorithm without lattice bias. *Journal of Physics: Condensed Matter* **2009**, *21* (8), 084204. DOI: 10.1088/0953-8984/21/8/084204.
(19) Nørskov, J. K.; Rossmeisl, J.; Logadottir, A.; Lindqvist, L.; Kitchin, J. R.; Bligaard, T.; Jónsson, H. Origin of the Overpotential for Oxygen Reduction at a Fuel-Cell Cathode. *The Journal of Physical Chemistry B* **2004**, *108* (46), 17886–17892. DOI: 10.1021/jp047349j.
